# P2RX7 Purinoceptor: A Therapeutic Target for Ameliorating the Symptoms of Duchenne Muscular Dystrophy

**DOI:** 10.1371/journal.pmed.1001888

**Published:** 2015-10-13

**Authors:** Anthony Sinadinos, Christopher N. J. Young, Rasha Al-Khalidi, Anna Teti, Paweł Kalinski, Shafini Mohamad, Léonore Floriot, Tiphaine Henry, Gianluca Tozzi, Taiwen Jiang, Olivier Wurtz, Alexis Lefebvre, Mikhail Shugay, Jie Tong, David Vaudry, Stephen Arkle, Jean-Claude doRego, Dariusz C. Górecki

**Affiliations:** 1 Molecular Medicine, School of Pharmacy and Biomedical Sciences, University of Portsmouth, Portsmouth, United Kingdom; 2 Department of Biotechnological and Applied Clinical Sciences, University of L’Aquila, L’Aquila, Italy; 3 Departments of Surgery, Immunology, and Bioengineering, School of Medicine, University of Pittsburgh, Pittsburg, Pennsylvania, United States of America; 4 School of Engineering, University of Portsmouth, Portsmouth, United Kingdom; 5 Platform of Behavioural Analysis (SCAC), University of Rouen, Mont-Saint-Aignan,Rouen, France; 6 INSERM U982, Plate-Forme d’Imagerie PRIMACEN, IRIB, University of Rouen, Mont-Saint-Aignan, France; 7 Genomics of Adaptive Immunity Lab, Shemyakin and Ovchinnikov Institute of Bioorganic Chemistry and Pirogov Russian National Research Medical University, Moscow, Russia; 8 National Center of Scientific Research (CNRS), Caen, France; Stanford University, UNITED STATES

## Abstract

**Background:**

Duchenne muscular dystrophy (DMD) is the most common inherited muscle disease, leading to severe disability and death in young men. Death is caused by the progressive degeneration of striated muscles aggravated by sterile inflammation. The pleiotropic effects of the mutant gene also include cognitive and behavioral impairments and low bone density.

Current interventions in DMD are palliative only as no treatment improves the long-term outcome. Therefore, approaches with a translational potential should be investigated, and key abnormalities downstream from the absence of the DMD product, dystrophin, appear to be strong therapeutic targets. We and others have demonstrated that DMD mutations alter ATP signaling and have identified P2RX7 purinoceptor up-regulation as being responsible for the death of muscles in the *mdx* mouse model of DMD and human DMD lymphoblasts. Moreover, the ATP–P2RX7 axis, being a crucial activator of innate immune responses, can contribute to DMD pathology by stimulating chronic inflammation. We investigated whether ablation of *P2RX7* attenuates the DMD model mouse phenotype to assess receptor suitability as a therapeutic target.

**Methods and Findings:**

Using a combination of molecular, histological, and biochemical methods and behavioral analyses in vivo we demonstrate, to our knowledge for the first time, that genetic ablation of *P2RX7* in the DMD model mouse produces a widespread functional attenuation of both muscle and non-muscle symptoms. In dystrophic muscles at 4 wk there was an evident recovery in key functional and molecular parameters such as improved muscle structure (minimum Feret diameter, *p <* 0.001), increased muscle strength in vitro (*p* < 0.001) and in vivo (*p* = 0.012), and pro-fibrotic molecular signatures. Serum creatine kinase (CK) levels were lower (*p* = 0.025), and reduced cognitive impairment (*p* = 0.006) and bone structure alterations (*p <* 0.001) were also apparent. Reduction of inflammation and fibrosis persisted at 20 mo in leg (*p* = 0.038), diaphragm (*p* = 0.042), and heart muscles (*p <* 0.001). We show that the amelioration of symptoms was proportional to the extent of receptor depletion and that improvements were observed following administration of two P2RX7 antagonists (CK, *p* = 0.030 and *p* = 0.050) without any detectable side effects. However, approaches successful in animal models still need to be proved effective in clinical practice.

**Conclusions:**

These results are, to our knowledge, the first to establish that a single treatment can improve muscle function both short and long term and also correct cognitive impairment and bone loss in DMD model mice. The wide-ranging improvements reflect the convergence of P2RX7 ablation on multiple disease mechanisms affecting skeletal and cardiac muscles, inflammatory cells, brain, and bone. Given the impact of P2RX7 blockade in the DMD mouse model, this receptor is an attractive target for translational research: existing drugs with established safety records could potentially be repurposed for treatment of this lethal disease.

## Abstract 2

Dariusz Gorecki and colleagues investigate the effect of P2RX7 ablation on muscle, brain, and bone pathology in a DMD model mouse.

## Editors' Summary

**Background:**

Muscular dystrophies are inherited diseases in which the body’s muscles gradually weaken and waste away. The most common and severe muscular dystrophy—Duchenne muscular dystrophy (DMD)—also includes cognitive (thinking) and behavioral impairments and low bone density as well as chronic inflammation. DMD affects about 1 in 3,500 boys; girls can be carriers of DMD but rarely have any symptoms. At birth, boys who carry a mutation (genetic change) in the gene that makes the protein dystrophin seem normal, but the symptoms of DMD soon begin to appear. Affected children may initially have difficulty walking or may find it hard to sit or stand independently. As they age, their muscle strength progressively declines, a process that is aggravated by sterile inflammation (an immune system response to tissue damage that occurs in the absence of an infectious agent), and most affected boys are confined to a wheelchair by the time they are 12 years old. The diaphragm and other muscles involved in breathing also weaken, and the heart muscle becomes enlarged. Consequently, few boys with DMD live beyond their early 20s, usually dying from breathing or heart problems. At present, there is no cure for DMD. However, physical therapy and treatment with steroids (intended to reduce sterile inflammation) can prolong the ability of patients to walk, and assisted ventilation can help with their breathing.

**Why Was This Study Done?:**

One way to treat DMD under investigation is replacement of the defective dystrophin in muscles using gene therapy. Dystrophin normally forms structural scaffolds that sit in the membranes that surround muscle fibers and protect the fibers from damage during muscle contraction. In DMD, the loss of dystrophin, dystrophin-associated proteins, and specific signaling processes causes progressive muscle loss. Although gene therapy approaches that target dystrophin hold some promise, achieving sufficient dystrophin expression in all the crucial muscle groups to prevent progressive muscle damage is hard. Moreover, gene therapy targeted at muscles will not treat the non-muscle-related characteristics of DMD. Targeting an abnormality downstream of dystrophin might therefore be a better approach to the treatment of DMD. One such target is P2RX7. This purinoceptor was originally identified as a sensor of ATP released from damaged cells and is an activator of innate immune responses. Because upregulation of P2RX7 is responsible for muscle death in the *mdx* mouse model of DMD and for the death of human DMD lymphoblasts, in this study, the researchers investigate whether genetic ablation of P2RX7 can attenuate the DMD symptoms of the *mdx* mouse model.

**What Did the Researchers Do and Find?:**

The researchers mated *mdx* mice and mice that lack the gene for P2RX7 to obtain Pf-*mdx*/P2RX7^−/−^ mice, which make no functional dystrophin or P2RX7. They then compared the structure and function of the muscles (dystrophic pathology) in these mice with those in *mdx* mice. They also examined specific aspects of the behavior of the mice. At four weeks, there was improved muscle structure and strength, decreased inflammation, and decreased fibrosis (thickening and scarring of the connective tissue covering the muscles) in the Pf-*mdx*/P2RX7^−/−^ mice compared to the *mdx* mice. P2RX7 ablation also reduced blood levels of creatinine kinase (a marker of muscle, heart, and brain injury), cognitive impairment, and bone structure alterations. Importantly, the reduction in inflammation and fibrosis was still evident at 20 months in the leg, diaphragm, and heart muscles of the Pf-*mdx*/P2RX7^−/−^ mice compared to the *mdx* mice. Finally, the dystrophic pathology in *mdx* mice could also be reduced by treating these mice with P2RX7 antagonists (molecules that bind to P2RX7 and prevent its function).

**What Do These Findings Mean?:**

These findings show that genetic ablation of P2RX7 can improve muscle function in the short and long term and can also correct cognitive impairment and bone loss in a mouse model of DMD. Thus, in *mdx* mice, P2RX7 ablation affects multiple disease mechanisms that affect skeletal and heart muscles, inflammatory cells, brain, and bone. Other preliminary findings suggest that P2RX7 blockade in *mdx* mice also improves DMD symptoms. These are promising results, but results in animals do not necessarily translate into effective clinical treatments. Nevertheless, these findings identify P2RX7 as an attractive target for the treatment of DMD, particularly since it might be possible to repurpose P2RX7 antagonists originally developed for the treatment of chronic pain for the treatment of DMD.

**Additional Information:**

This list of resources contains links that can be accessed when viewing the PDF on a device or via the online version of the article at http://dx.doi.org/10.1371/journal.pmed.1001888.

- The US National Institute of Neurological Disorders and Stroke provides information on muscular dystrophy (in English and Spanish)
- The US National Human Genome Research Institute also provides basic information on Duchenne muscular dystrophy and links to additional resources
- The US Centers for Disease Control and Prevention has information about muscular dystrophy
- The not-for-profit Nemours Foundation provides information about muscular dystrophy for parents, children, and teenagers (in English and Spanish)
- The US not-for-profit organization Parent Project Muscular Dystrophy provides detailed information about all aspects of Duchenne muscular dystrophy and parents’ stories about Duchenne muscular dystrophy
- MedlinePlus provides links to further resources on muscular dystrophy and an encyclopedia page on Duchenne muscular dystrophy (in English and Spanish)
- Wikipedia has pages about Duchenne muscular dystrophy and P2RX7 (note that Wikipedia is a free online encyclopedia that anyone can edit; available in several languages)
- TREAT-NMD is a network for the neuromuscular field that provides an infrastructure to ensure that the most promising new therapies reach patients as quickly as possible

## Introduction

Duchenne muscular dystrophy (DMD) results in loss of dystrophin, which disrupts structural scaffolds for dystrophin-associated proteins (DAPs) as well as specific signaling processes, causing progressive muscle loss with sterile inflammation [1]. Symptoms also include cognitive and behavioral impairment [2] and bone structure abnormalities [3], both irrespective of the functional muscle impairment. This symptom diversity illustrates the importance of DMD gene expression in various cells.

Molecular approaches aimed at restoration of dystrophin hold some promise, but achieving the 15%–20% level of expression required to fully protect muscle fibers [4] in all crucial muscle groups remains a challenge. Moreover, muscle targeting would not tackle non-muscle symptoms. Therefore, alternative strategies should be investigated, and treatments aimed at alterations downstream from the absence of dystrophin have shown therapeutic promise [5]. Clearly, targeting signaling pathways using pharmacological agents is currently more achievable than restoration of structural proteins via molecular approaches.

We and others have demonstrated that DMD mutations impact on the control of ATP signaling and have identified P2RX7 up-regulation as being responsible for the death of human DMD lymphoblasts and muscles in the *mdx* mouse model of DMD [6–11]. Analyzing the consequences of P2RX7 activation, we discovered a novel mechanism of autophagic cell death, and pharmacological blockade or genetic ablation of P2RX7 proved protective against the ATP-induced death of dystrophic muscles [12].

P2RX7 belongs to a family of cell membrane ATP-gated ion channels. Unlike some other purinoceptors, full activation of P2RX7 requires high levels of extracellular ATP (eATP), which occur in inflammatory conditions [13]. P2RX7 was originally identified on macrophages and lymphocytes as a sensor of eATP released from damaged cells (one of the damage/danger-associated molecular patterns [DAMPs]) and was considered an activator of the “danger mode” of the immune response [14].

Levels of cytoplasmic ATP in skeletal muscles are particularly high, so there is a potential for 5–10 mM eATP at the dystrophic cell membrane [10]. Furthermore, the DAP α-sarcoglycan is a muscle-specific ATP hydrolase responsible for 25% of eATP degradation [15]. In DMD, α-sarcoglycan is lost from the sarcolemma [1], which raises eATP levels, creating an environment consistent with increased activation of P2RX7.

Crucially, P2RX7 expression and function are increased in many diverse pathologies such as rheumatoid arthritis, graft-versus-host disease, transplant rejection, neuro-inflammation, pain [16], and limb-girdle muscular dystrophy type 2B—another muscular dystrophy with an inflammatory component [8]. As P2RX7 upregulation is present in both DMD [6] and *mdx* mice [9], over-activation of P2RX7 could contribute to damage both directly by causing the death of dystrophic muscles [12] and indirectly by stimulating harmful inflammatory responses.

**Fig 1:**
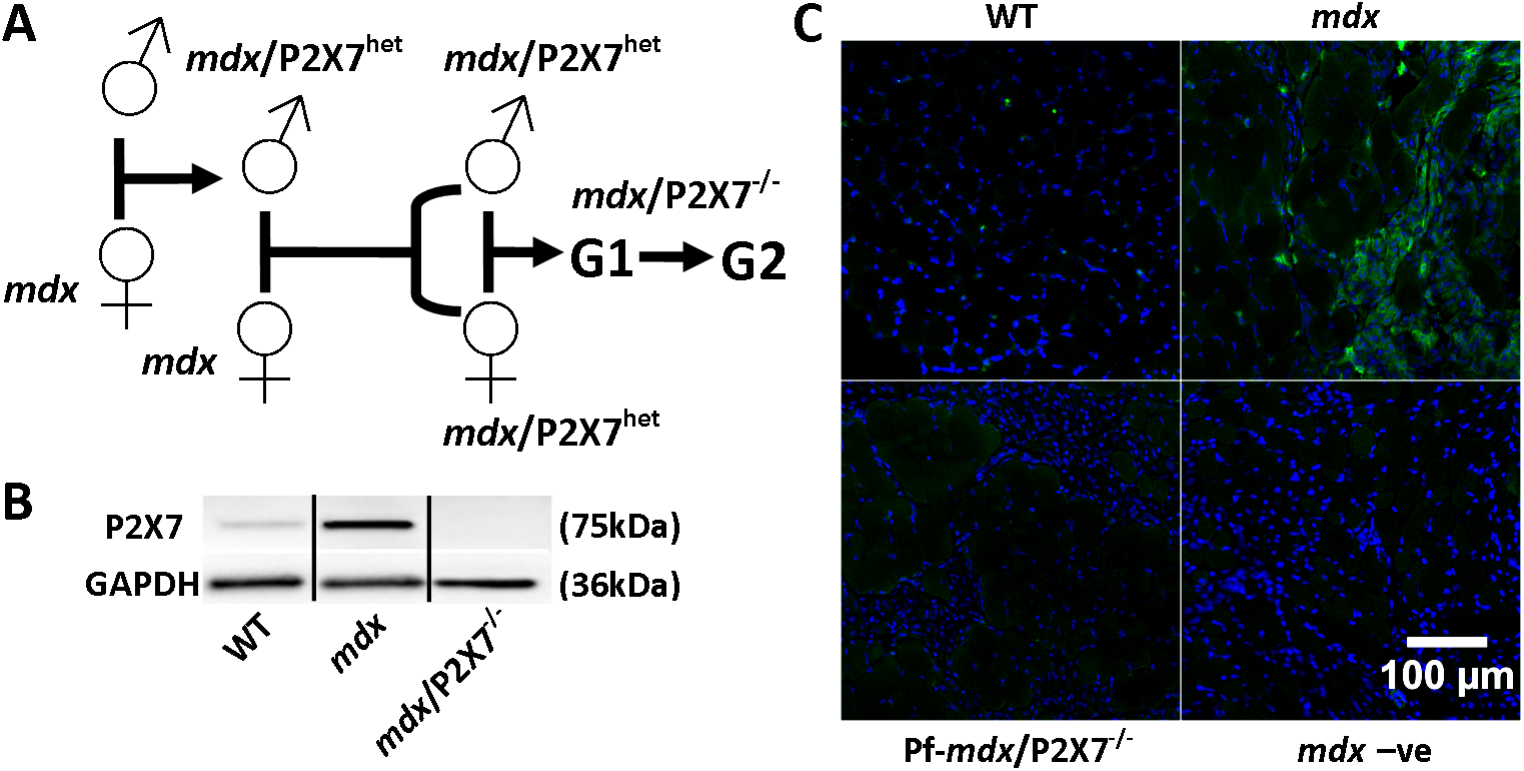
Generation and characterization of *mdx*/P2X7^−/−^ mice. (A) Schematics of mouse breeding. (B) Representative Western blots showing increased expression of P2RX7 in 4-wk-old *mdx* compared to wild-type (WT) gastrocnemius and its absence in *mdx*/P2X7^−/−^. Use of separate Western blots is indicated by solid black lines. (C) Micrographs of P2RX7 immunofluorescence localization (green signal) in 4-wk-old tibialis anterior (TA) muscle from WT, *mdx*, and Pf-*mdx*/P2X7^−/−^ mice showing expression in areas rich with infiltrating cells, and negative control using no primary antibody and with a blue signal denoting nuclear counterstaining.

We bred *mdx* mice with Pfizer or Glaxo *P2RX7* knockout mice and developed two lines of dystrophic mice (Fig 1A). One line (Pf-*mdx*/P2X7^−/−^) lacked functional receptors [17]. The second (G-*mdx*/P2X7^−/−^) was an isoform knockout, in which the main (P2RX7a) variant was knocked out but the low-expression P2RX7k variant escaped inactivation [18]. Therefore, the latter served as an additional control. These models were used to establish the impact of *P2RX7* ablation on the dystrophic pathology in muscle, brain, and bone and to assess its suitability as a therapeutic target.

## Methods

All animal experiments were performed in accordance with approvals from the Institutional Ethical Review Board, University of Portsmouth, and the Home Office, UK (70/7479), and with the recommendations of Directive 2010/63/EU of the European Parliament and of the Council of September 22, 2010, L276/33 (20.10.2010). The protocols (see below and S1 Checklist) were approved by the French Local Ethical Committee of Normandy (CENOMEXA; approval numbers N/14-04-04-16, N/15-04-04-17, and N/16-04-04-18) and were conducted in an authorized animal facility (authorization number C-76-540-2), under the supervision of authorized investigators (J. C. do Rego; authorization number 76.A.29 from the Ministère de l’Agriculture et de la Pêche, France). Investigators were blinded to the sample group allocation, where possible.

### Generation of Double-Mutant Mice

Animals were central to this project, as there is no in vitro system allowing testing of a disease involving muscle damage and inflammatory processes, which are, in addition, interdependent. In a consensus opinion of experts, the *mdx* mouse is currently the most appropriate preclinical model to test treatment efficacy for DMD (http://www.treat-nmd.eu/research/preclinical/dmd-sops/).

Pfizer and Glaxo *P2RX7* knockout male mice [19,20] were crossed with *mdx* C57Bl/10ScSn-Dmdmdx/J female mice (Harlan Laboratories). Male F1 mice were sequentially intercrossed with *mdx* females to generate double-mutant mice. The F3 generation showed the expected Mendelian distribution of genotypes, all of them on an *mdx* background. The P2X7^−/−^ and *mdx* genotypes were identified by PCR using ear biopsy genomic DNA. The genotype was confirmed by Western blotting of muscle samples, using anti-dystrophin and anti-P2RX7 antibodies (listed below).

To mimic the clinically relevant situation, analyses were performed in male mice (unbiased by gender). At 4 wk, *mdx* mice show muscle degeneration and regeneration and an inflammatory cell infiltration pattern akin to the human pathology. The diaphragm is the *mdx* muscle that undergoes progressive degeneration, which is particularly noticeable at older ages [21]. In DMD patients, those who survive to the third decade present with cardiomyopathy and heart failure [22]. Therefore, diaphragms and hearts were studied at 20 mo to investigate the late-stage effects of receptor ablation.

### Antagonist Administration

Starting at 2 wk of age, *mdx* mice were treated daily by intraperitoneal injection for 4 wk with 125 mg/kg body weight Coomassie Brilliant Blue G 250 (CBB) (Sigma-Aldrich) or for 4 wk with 8.4 mg/kg body weight oxidized ATP (ox-ATP) (Tocris Bioscience) or for 2 wk with 50 mg/kg body weight A-438079 (Tocris Bioscience). Dosage was based on previous studies [9,23,24]. Age-matched control mice received the same volume of sterile saline (for CBB and ox-ATP) or saline with 20% v/v DMSO solution (for A-438079).

### PCR Analysis

PCRs for the identification of *P2RX7*, WT, and mutant alleles were performed using the following primers: Pfizer exon-13 WT (Fv 5′-TGGACTTCTCCGACCTGTCT; Rv 5′-TGGCATAGCACCTGTAAGCA) and neomycin cassette KO (Fv 5′-CTTGGGTGGAGAGGCTATTC; Rv 5′-AGGTGAGATGACAGGAGATC), and Glaxo exon-1 WT (Fv 5′-TGCCCATCTTCTGAACAC; Rv 5′-CTTCCTCTTACTGTTTCCTCCC) and LacZ KO variant (Fv 5′- TGCCCATCTTCTGAACAC; Rv 5′-GCAAGGCGATTAAGTTGGG). The *mdx* mutation was identified using ARMS (amplification refractory mutation system) primers as previously described [25]. PCR conditions were as follows: denaturing at 94°C for 3 min; 35–40 cycles of 94°C for 30–40 s, 55–59°C annealing for 30–40 s, and elongation at 72°C for 1 min per kilobase of expected PCR product length; followed by a final 10-min elongation step at 72°C.

### Quantitative PCR Analysis

Total RNA was extracted using the RNeasy Kit (Qiagen) per manufacturer’s instructions, and 1 μg of RNA was used for cDNA synthesis. 25–50 ng of the cDNA was used to perform expression analyses with Taqman on the StepOnePlus System (Applied Biosystems) or using the ViiA 7 Real-Time PCR System (Life Technology) and using SYBR Green on the LightCycler 1536 (Roche). All data were analyzed using Qbase^+^ software (Biogazelle). Quantitative PCR (qPCR) primers used for the analysis were purchased from Applied Biosystems or Primer Design. A set of 12 candidate reference genes (Primer Design) was tested, and the most stably expressed genes were determined using the geNorm tool of the Qbase^+^ software. These were used as reference to establish individual gene expression values (2^−ΔΔCT^). Four to five biological replicates (individual mice) for each genotype were analyzed in duplicate, and all samples were run on the same plate to avoid inter-run variation and calibration. qPCR analyses were performed in Portsmouth, Rouen, and Pittsburgh, and results for overlapping transcripts were cross-referenced between collaborating centers.

### RNA-Seq Analysis

RNA was extracted as described above from TA muscles isolated from four individual mice per genotype. RNA quality was confirmed using Bioanalyzer (Agilent) per manufacturer’s instructions, and RNA-Seq was performed by a commercial service provider, Source BioScience. Raw FASTQ files were preprocessed by quality and adapter trimming and mapping to mouse genome (mm10 genome assembly, UCSC Genome Browser), and expression quantification was performed using the Tuxedo suite (TopHat and Cufflinks, [http://www.nature.com/nprot/journal/v7/n3/full/nprot.2012.016.html]). Genes of interest were selected to match the Qiagen mouse fibrosis PCR array (http://www.sabiosciences.com/rt_pcr_product/HTML/PAMM-120A.html). The resulting gene-level expression table for relevant RNAs is available as S2 Table.

### Antibodies

The following antibodies were used: CD4–14–0041, CD8–14–0081, and Ly6G—14–5931, all rat monoclonal (eBioscience); IL1B—8689, rabbit polyclonal (Cell Signaling Technology); F4/80—ab74383 and collagen type-Ia—ab34710, both rabbit polyclonal (Abcam); CD68—MCA1957GA and CD206—MCA2235GA, both rat monoclonal (AbD Serotec); CD163—sc-33560, rabbit polyclonal (Santa Cruz Biotechnology); dystrophin—MANDRA1, mouse monoclonal, and myogenin—FD5, rat monoclonal (Developmental Studies Hybridoma Bank); LC3II—L7543 and actin—A2066, both rabbit polyclonal, and GAPDH—G9545 (Sigma-Aldrich); collagen type-IV—AB769, goat polyclonal (Chemicon); P2X7–177 003, rabbit polyclonal (Synaptic Systems); β-tubulin—IMG-5810A, rabbit polyclonal (Imgenex); dystrophin—2166, rabbit polyclonal, was a kind gift from D. J. Blake, Cardiff University.

### Western Blotting

Total proteins from frozen tissues were extracted by crushing samples in liquid nitrogen, with further homogenization in extraction buffer: 1× Lysis-M, 1× protease inhibitor cocktail, 2× phosphatase inhibitor cocktail (all Roche), 2 mM sodium orthovanadate (Sigma-Aldrich). All samples were centrifuged (800*g* for 3 min at 4°C), and protein concentrations were determined using the Bicinchoninic Acid Kit (Sigma-Aldrich). 20–40 μg of protein was mixed with Laemmli buffer at a 1:1 v/v ratio with 5% v/v β-mercaptoethanol, heated for 5 min at 95°C, and chilled on ice. Samples were separated on 6%–12% w/v SDS-polyacrylamide gels and electroblotted onto Hybond C membranes (Amersham). Blots were blocked in 5% w/v nonfat milk powder in 1× Tris buffered saline with Tween 20 (TBST) (0.01% v/v Tween 20; Sigma-Aldrich) for 1 h prior to probing with a primary antibody diluted in the same blocking buffer (overnight at 4°C or for 2 h at room temperature), then blots were washed (3×) with 1× TBST for 10 min and incubated with the appropriate horseradish-peroxidase-conjugated secondary antibody (Sigma-Aldrich) for 45 min. Specific protein bands were visualized using luminol-based substrates (Uptilight US, Cheshire Sciences), and images were obtained using a G:BOX Chemi XT16 system (Syngene). β-actin, GAPDH, and tubulin were used as protein-loading controls. All densitometric analyses of specific protein bands were made using exposure times within the linear range and the integrated density measurement function of ImageJ software [26,27].

### Immunolocalization

Frozen muscle was transferred to a cryostat chamber and allowed to equilibrate to −20°C. Cryosections 5- to 10-μm thick were then cut from the middle third of the sample and collected on poly-L-lysine (0.5 mg/ml)–coated glass slides. Sections were allowed to air dry for several hours. Samples were fixed in a 2%–4% w/v paraformaldehyde solution in TBST for 15 min at 4°C, followed by two washes in TBST. The primary antibody incubation in TBST containing 10% v/v serum was applied for 2 h at room temperature or overnight at 4°C. Three 5-min TBST washes were applied before secondary antibody incubation in TBST and 2% v/v serum containing Hoechst fluorescent nuclear counterstain for 1 h at room temperature. Sections were finally washed three times for 10 min before mounting in FluorSave (Merk Millipore) fluorescence mounting medium. Either entire cross-sections through the mid-portion of TA muscles were captured in their entirety using a zoom microscope (Axiozoom V.16, Zeiss), or whole cross-sections were made of montaged 20× magnification fields of view. For quantification of immunofluorescent cells, a semi-automated (unbiased) method using a thresholding macro designed in ImageJ was used. Numbers were then expressed per unit of area. For diaphragms, counts per unit of area for each animal were derived by averaging the counts from five fields of view encompassing a significant portion of each diaphragm cross-section. Counts were also made using the threshold and analyze particles functions of ImageJ. Enumeration of dystrophin revertant fibers (the dystrophin-expressing fibers arising due to spontaneous exon skipping occurring in myogenic cells) was achieved using the cell counter plugin of ImageJ [26,27] applied to whole cross-sections of diaphragm. Revertant fibers were reported as number per square millimeter. For the quantification of IgG permeability into muscle fibers, the entire cross-section was analyzed. Manually delineated IgG-positive (compromised) fluorescent areas were compared (percent of total area of the muscle cross-section).

### Morphometric Analysis

Muscle fiber size and central nucleation were visualized by collagen type-IV and Hoechst immunofluorescence staining of frozen muscle sections. Individual microscope fields of view were montaged using ImageJ to present whole cross-sections through the muscle. Image analysis was performed on these composite images using Fiji and ImageJ open-source software (US National Institutes of Health). A macro was developed to sequentially (i) subtract background components to minimize noise that could interfere with further analysis; (ii) apply band-pass thresholds to separate color channels; (iii) dilate borders to close inconsistent gaps; (iv) skeletonize these borders; (v) apply a convolution filter to translate pixels uniformly for border detection; (v) generate a mask of the muscle fiber borders using the analyze particles function, simultaneously eliminating stray “non-border” signals; and (vi) overlay threshold-delimited nuclei over the border mask, before another analyze particles command was used to measure morphometric variables including “area” and “minimum Feret diameter.” Fiji image processing steps and macro construction were partially derived from published standard operating procedures [28] (http://treat-nmd.eu/research/preclinical/dmd-sops/). An average of 3,243 muscle fibers per 4-wk-old TA were measured: the *mdx* TA had an average of 3,161 fibers and the Pf-*mdx*/P2X7^−/−^ TA had an average of 3,325 fibers. In the 20-mo-old TA analysis, an average of 4,639 muscle fibers were measured per cross-section: *mdx* TA had an average of 4,493 fibers and Pf-*mdx*/P2X7^−/−^ TA had an average of 4,786 fibers. There was no difference in the number of muscle fibers between cross-sections from *mdx* and Pf-*mdx*/P2X7^−/−^ TA (*t*-test, *t* = 0.51, df = 7, *p* = 0.624). Fiber number per unit of tissue area is presented. The step-by-step protocols developed by us and used in this study are shown as video available at http://youtu.be/GZVaRQYgGQU (morphometric analysis using Fiji) and http://youtu.be/oxyM7r7VYp0 (central nucleation count from the analyze particles output).

### Trichrome Staining

The trichrome method employed here is the TREAT-NMD-recommended protocol (http://treat-nmd.eu/research/preclinical/dmd-sops/). Briefly, 10-μm-thick cryosections were fixed in 4% w/v PFA and 0.1 M PBS followed by Bouin’s fixative, stained with Biebrich scarlet-acid fuchsin solution (Sigma-HT151, Sigma-Aldrich) for 20 min, washed in water, and incubated in a phosphotungstic/phosphomolybdic acid solution (5% w/v phosphotungstic acid, 5% w/v phosphomolybdic acid, DDH_2_O) for 2 × 3 min, directly before incubation in aniline blue solution (2.5% w/v aniline blue, 2% v/v acetic acid, DDH_2_O) for 7 min. Optionally, sections were washed in water and incubated in glacial acetic acid solution (1% v/v; DDH_2_O) for 1 min. Finally, sections were washed, dehydrated, and mounted, and the entire sections were visualized using bright-field Axiozoom V.16 (Zeiss); positive (blue) areas are expressed as percent of the total area of each muscle cross-section.

### Serum Creatine Kinase Levels

Sera were prepared and creatine kinase (CK) levels were analyzed using the Creatine Kinase Enzymatic Assay Kit (3460–07, BioScientific) or the Creatine Kinase Activity Assay Kit (Mak116-1kt, Sigma-Aldrich), according to manufacturers’ instructions.

### Bone Morphometric Analyses (6 mo)

Bones were removed, cleared of soft tissues, and analyzed by micro-computed tomography (μCT) as described previously [3]. Briefly, μCT 40 (Scanco Medical) was used to assess trabecular bone volume fraction (bone volume/total volume [BV/TV]), micro-architecture in the metaphyseal region of the tibia, and cortical geometry at the mid-tibia. For trabecular bone, BV/TV (percent), trabecular thickness, trabecular number (the number of plates per millimeter of length), and trabecular separation (Tb.Sp) (micrometers) were assessed on 100 contiguous μCT slides, starting 100 slides below the growth plate.

### Bone Morphometric Analyses (4 wk)

Mice tibias were imaged by means of μCT (XT H 225, X-Tek Systems), and a complete data acquisition was performed (V = 50–55 kV, I = 95–110 μA, voxel size = 6–8 μm, rotational step = 0.19°/360°, acquisition time = 90 min). The regions of interest used for the morphometric assessment were selected to include both the cancellous and cortical bones in the proximal tibias and the cortical bones in the mid-shaft of the tibias, similarly to the methodology used in [3]. Image processing was performed using the seeded region growing (SRG) technique (ImageJ) to automate segmentation of the bone tissue from the background. The morphometric parameters measured were bone volume fraction (BV/TV), trabecular bone thickness, and Tb.Sp.

### Force Measurements in Diaphragms Ex Vivo

Whole diaphragms were excised from 4-mo-old WT, *mdx*, Pf-*mdx*/P2X7^−/−^, and G-*mdx*/P2X7^−/−^ mice and placed into Krebs-Ringer solution (NaCl 118 mM, KCl 4.7 mM, NaHCO_3_ 24.88 mM, KH_2_PO_4_ 1.18 mM, glucose 11.1 mM, MgSO_4_ 0.82 mM, CaCl_2_ 2.52 mM), taking care not to handle the muscle wherever possible. Sutures were then tied to two points on either side of a segment of attached rib, which were then attached to an immobile plastic clamp. A centrally derived triangular section of the diaphragm was used for testing. Contractile force was translated along another suture, tied to the central tendon apex of the approximately equilateral triangular section of muscle. This suture was, in turn, attached to a mechanical force transducer (ADInstruments), amplifier, and data acquisition setup. Excitation of muscle was achieved via local field potentials through platinum electrodes in oxygenated (95% O_2_, 5% CO_2_) Krebs-Ringer solution, at a constantly maintained temperature of 37°C.

Each diaphragm was stretched in very small increments from an initial resting state to establish the optimal excitation-to-force generation length. Diagnostic testing also confirmed that the voltage twitch stimulus of 140 V (2-ms length) was adequate to elicit a maximal twitch response. To achieve a maximal isometric tetanic force response, diaphragm sections were subjected to a 140 V (2 ms) stimulus train at 100-Hz frequency for 0.5–1 s. The test regime involved collecting six twitch responses, followed by six tetanic trains, with a 2-min rest period between each. Maximal twitch and tetanic response were taken from the tallest of the respective force traces. All forces were normalized to muscle wet weight and expressed as Newtons per gram of tissue (N/g). This testing strategy [29] followed the TREAT-NMD standard operating procedures for the use of experimental animals (http://treat-nmd.eu/research/preclinical/dmd-sops/).

### Running Wheel Activity

Locomotor activity was assessed using cages equipped with voluntary running wheels (Intellibio), placed in a dimly lit, quiet, and ventilated room, at a temperature of 22 ± 1°C, under a 12 h light/12 h dark cycle (light on between 7:00 a.m. and 7:00 p.m.). Mice were individually housed in cages. The recording apparatus was connected to a computer to process the data. Animals had 24-h access to the wheels, and total distance travelled was measured.

### Grip Strength Test

In this and all other in vivo tests, investigators were blinded with respect to the sample group allocation.

The grip strength test was performed to assess muscular strength and forepaw grasping reflex. The mouse was held by the tail and slowly approached to a metallic grid (6 × 6 cm) connected to a force sensor (Bioseb). Once the animal gripped the grid by its forelimbs, a gentle horizontal traction was applied to the tail until the animal let the grid go. The maximal force was recorded over two trials with a 1-min inter-trial interval. Strength was estimated by the mean of both trials.

### Elevated Zero Maze Test

Anxiogenic activity was assessed in the elevated zero maze test, a pharmacologically validated assay of anxiety in rodents, based on the natural aversion of mice to elevated and open spaces. The apparatus consisted of an infrared-transmitting, ring-shaped, black Plexiglas platform (outer diameter 45 cm, width 6 cm), placed 60 cm above ground level in a dimly illuminated room, divided equally into four quadrants with two opposing open quadrants and two opposing closed quadrants (surrounded by a 27-cm wall from the surface of the maze). Each mouse, previously isolated for 15 min before the experiment in a small individual cage, was placed at the end of an open section, with its head facing a closed quadrant. The time spent and the distance travelled in open and closed quadrants during a 5-min period were recorded using the EthoVision XT 9.0 automated image analysis system (Noldus Information Technologies).

### Rotarod Test

The Rotamex-5 (Columbus Instruments) was used for the evaluation of motor coordination. Mice were placed onto the rod of the apparatus at 0 rpm to allow them to balance and then rotation was increased by about 0.4 rpm/s, to a maximum of 17 rpm. The time period for which the mice were able to maintain their balance on the bar was recorded automatically using photobeam break technology and the instrument’s software.

### Parallel Rod Floor Test

This test was performed using the parallel rod floor apparatus (Stoelting Europe) as described previously [30]. Briefly, the instrument floor consisted of a series of parallel steel rods, a stainless steel base plate with an acrylic border raised 1 cm above the base plate, and a clear acrylic box with no bottom and a removable lid. Locomotor activity and number of foot slips were recorded during two consecutive periods of 5 min. A slip was detected by the AnyMaze automated analysis system as a paw touched the base plate, completing a circuit.

### Novel Object Recognition Test

Experiments were undertaken using a Plexiglas open field box (40 × 40 cm) with grey walls (30 cm high) and a white floor under constant room temperature (23 ± 1°C) and homogeneous dim illumination (open field center: 40 lux). To test the memory retention of each mouse at three distinct retention intervals, three sets of three objects of different shapes and color were used, which were either glass, plastic, or metal. The procedure started with a 5-d habituation period [31]. The object recognition test started 2 d after the end of habituation. Mice were first submitted to a single acquisition trial, where they were exposed to two new identical objects for 10 min. Memory retention was tested at 10 min, 24 h, or 48 h. Each animal was thus submitted to three successive acquisition/retention phases, following a sequence of retention delays that was counterbalanced among individuals. During these test phases, one of the familiar objects was replaced by a novel object, with a different shape and color. During the 5-min testing, exploration of an object was defined as pointing the nose to the object and/or touching the object with the nose. The total time spent with each object and number of times each object was explored were recorded and scored using fully automated EthoVision XT 10.0 video tracking software (Noldus Information Technologies). Data are presented as the discrimination index (time or frequency exploring novel object × 100/total object exploration time or frequency).

### Statistical Analysis

As detailed in the figure legends, Student’s *t* unpaired test was used for comparisons between two data groups (P2X7 protein; CD4, CD8, and Ly6G/tubulin mRNA levels; CD68-positive and revertant fiber counts; CD11b and P2X4 protein; in vivo effects of P2X7 antagonists). All other statistical analyses were for three or more data groups and employed ANOVA with Tukey’s post hoc test. In order to account for possible heteroscedasticity of data, the Anderson-Darling normality test was applied to data that yielded *p <* 0.05 from ANOVA, and permutation analysis was applied using Treeperm v1.6 script in R Studio v3.2.2. With the exception of serum CK levels in Pf-*mdx*/P2X7^−/−^ mice and F4/80, Foxp3, and IL12 levels in dystrophic muscle (to which Mann-Whitney U and Kruskal-Wallis H tests were applied), Anderson-Darling tests yielded *p* > 0.05, and, unless otherwise stated, permutation analysis also yielded *p <* 0.05. A *p-*value of <0.05 was considered statistically significant, and the values are reported as follows in figures: *p < 0.05, **p < 0.005, ***p < 0.001.

## Results

### P2RX7 Ablation Improves *mdx* Mouse Muscle Structure and Function

**Fig 2:**
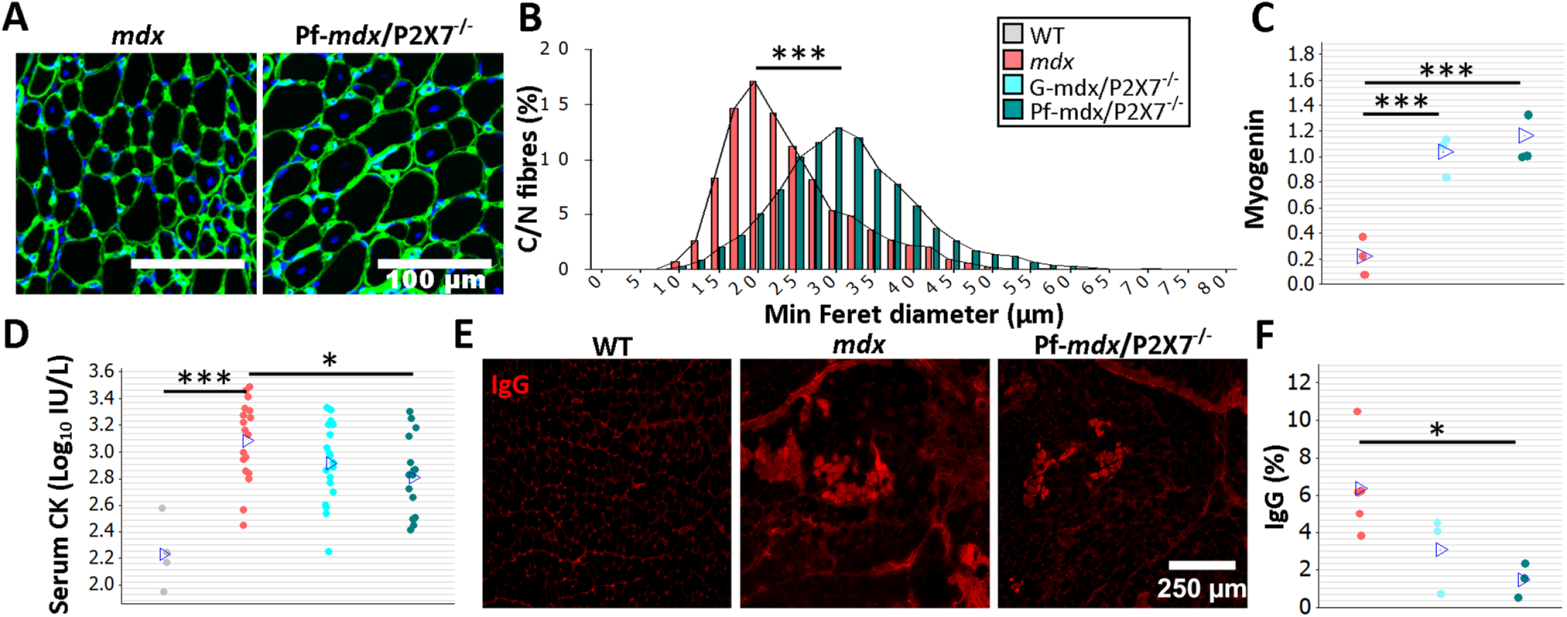
P2RX7 ablation reduces *mdx* mouse muscle pathology. The color-coding legend applies to all graphs in the figure. (A) Collagen type-IV (green) and nuclei (blue) immunofluorescence with an accompanying frequency histogram (B) of the minimum Feret diameter of C/N fibers from 4-wk-old *mdx* and Pf-*mdx*/P2X7^−/−^ mice showing the right shift in TA muscle fiber size corresponding with the greater average Feret diameter of Pf-*mdx*/P2X7^−/−^ fibers (*t*-test, *t* = 6.99, df = 6, *p* < 0.001). (C) Elevated myogenin levels (average Western blot values) in both Pf-*mdx*/P2X7^−/−^ and G-*mdx*/P2X7^−/−^ muscle (ANOVA, *F* = 33.38, df = 2, *n* = 4, 3, 4, *p <* 0.001; Tukey’s test, G-*mdx*/P2X7^−/−^ versus *mdx*, *p* < 0.001; G-*mdx*/P2X7^−/−^ versus Pf-*mdx*/P2X7^−/−^, *p* = 0.516; Pf-*mdx*/P2X7^−/−^ versus *mdx*, *p* < 0.001) and (D) significantly lower average serum CK levels in Pf-*mdx*/P2X7^−/−^ compared to *mdx* muscle (Mann-Whitney U test, *W* = 388, *n* = 18, 16, *p* = 0.012; permutation analysis, *F* = 7.07, *p* = 0.013; log_10_ serum CK ANOVA, *F* = 3.76, df = 2, *n* = 19, 18, 16, *p* = 0.030; Tukey’s test, Pf-*mdx*/P2X7^−/−^ versus *mdx*, *p* = 0.025). (E) Example immunofluorescence micrographs of IgG penetration into muscle fibers and (F) chart showing reduced average IgG influx into Pf-*mdx*/P2X7^−/−^ muscle (ANOVA, *F* = 5.52, df = 2, *n* = 3, 5, 3, *p* = 0.031; Tukey’s test, Pf-*mdx*/P2X7^−/−^ versus *mdx*, *p* = 0.032). *p < 0.05, ***p < 0.001.

To mimic the clinically relevant situation, we analyzed the effects of *P2RX7* ablation in 4-wk-old male mice. At this age, *mdx* limb muscles showed significantly increased expression of P2RX7 (Fig 1B and 1C) and the typical degeneration and regeneration pattern akin to human pathology. Gross histological features of *mdx* and *mdx*/P2X7^−/−^ muscles were similar, and the fraction of muscle fibers with centralized nuclei was not significantly different. However, using a semi-automated measurement of the minimum Feret diameter of muscle cross-sections, we found a significantly increased diameter of centrally nucleated (C/N) fibers (*p* < 0.001) in the Pf-*mdx*/P2X7^−/−^ TA (Fig 2A and 2B), and such an increase is a feature observed in less-severe phenotypes.

While mRNA levels of MyoD were similarly elevated in all dystrophic mice (S1 Table), transcript and protein levels of myogenin were higher in both *mdx*/P2X7^−/−^ strains compared to *mdx* (Fig 2C; G-*mdx*/P2X7^−/−^ versus *mdx*, *p* < 0.001; G-*mdx*/P2X7^−/−^ versus Pf-*mdx*/P2X7^−/−^, *p* = 0.516; Pf-*mdx*/P2X7^−/−^ versus *mdx*, *p* < 0.001), suggesting improved muscle regeneration.

Analyses of serum CK revealed significantly lower levels in 4-wk-old Pf-*mdx*/P2X7^−/−^ than in *mdx* mice (Fig 2D; untransformed Pf-*mdx*/P2X7^−/−^ versus *mdx* data, *p* = 0.013; log_10_ serum CK, *p* = 0.025), indicative of less sarcolemma damage and therefore less leakage of this intracellular muscle enzyme. This was confirmed by decreased sarcolemma permeability to blood-born IgG molecules [32] in *mdx*/P2X7^−/−^ muscle fibers (Fig 2E and 2F; Pf-*mdx*/P2X7^−/−^ versus *mdx*, *p* = 0.032).

**Fig 3:**
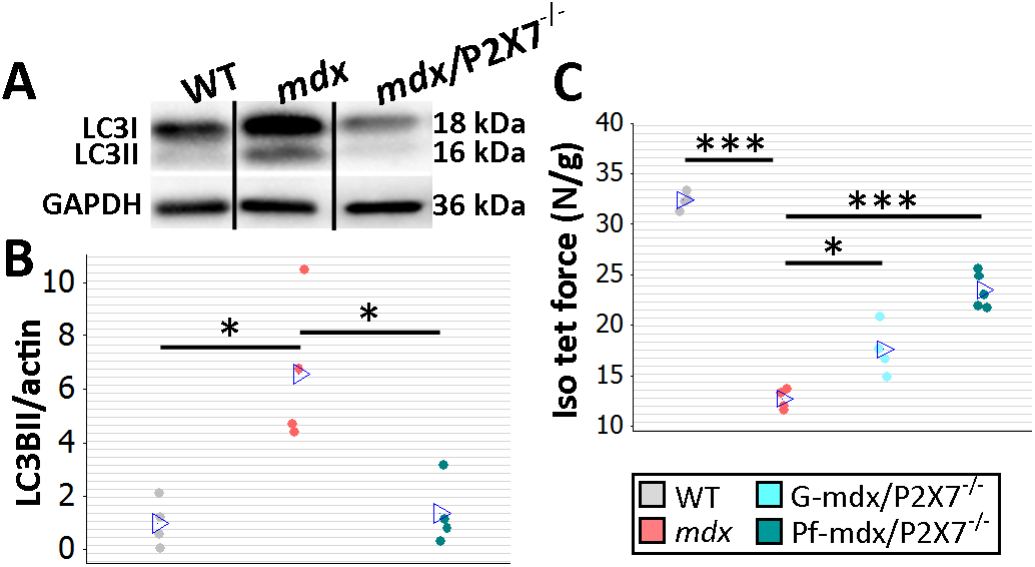
*P2RX7* ablation improves *mdx* mouse muscles. (A) Autophagy induction (LC3I to LC3II shift in representative Western blots) found in *mdx* muscles is blocked in Pf-*mdx*/P2X7^−/−^ muscles, with average values shown in (B) (ANOVA, *F* = 11.57, df = 2, *n* = 4, *p* = 0.003; Tukey’s test, Pf-*mdx*/P2X7^−/−^ versus *mdx*, *p* = 0.008). Note: use of separate Western blots is indicated by solid black lines. (C) Greater average diaphragm isometric tetanic forces at 4 mo in both Pf-*mdx*/P2X7^−/−^ and G-*mdx*/P2X7^−/−^ compared to *mdx* mice (ANOVA, *F* = 37.97, df = 2, *n* = 4, 4, 5, *p <* 0.001; Tukey’s test, G-*mdx*/P2X7^−/−^ versus *mdx*, *p* = 0.010; Pf-*mdx*/P2X7^−/−^ versus *mdx*, *p <* 0.001). *p < 0.05, ***p < 0.001.

Moreover, the significantly reduced autophagy in 4-wk-old *mdx*/P2X7^−/−^ muscles in vivo (Fig 3A and 3B), indicated by decreased LC3II (Pf-*mdx*/P2X7^−/−^ versus *mdx*, *p* = 0.008), corresponded with protective effects of P2RX7 ablation against ATP-induced autophagic muscle death, previously shown in vitro [12].

Finally, we compared muscle force in diaphragm organ bath preparations: isometric tetanic forces generated by 4-mo-old dystrophic diaphragms with ablated *P2RX7* were significantly (approx. 30%–50%) greater (Pf-*mdx*/P2X7^−/−^ versus *mdx*, *p <* 0.001) than those generated by *mdx* muscles (Fig 3C).

Collectively, these data indicate that P2RX7 ablation resulted in significant amelioration of muscle pathology.

Analyses of G-*mdx*/P2X7^−/−^ mice, which retain the low-expression P2RX7k isoform [18], also in skeletal muscles [9], showed increased tetanic forces (Fig 3C; G-*mdx*/P2X7^−/−^ versus *mdx*, *p* = 0.010). Myogenin levels were elevated compared to *mdx* and in line with Pf-*mdx*/P2X7^−/−^ (Fig 2C; G-*mdx*/P2X7^−/−^ versus *mdx*, *p* < 0.001), while CK levels (Fig 2D; G-*mdx*/P2X7^−/−^ versus *mdx*, *p* = 0.111) and sarcolemma permeability to IgG, albeit lower, were not statistically significantly different. The intermediate effects seen in G-*mdx*/P2X7^−/−^ as compared to Pf-*mdx*/P2X7^−/−^ mice confirmed the association between decreased P2RX7 expression and the phenotypic improvements. Moreover, we analyzed F1 Pf-*mdx*/P2X7^+/−^ males. These heterozygous mice had intermediate levels of muscle P2RX7 expression (S1A Fig), exactly as shown before in immune cells [33]. Again, this intermediate level corresponded with the intermediate Feret diameter values in these mice (S1B Fig). F1 and G-*mdx*/P2X7^−/−^ data also confirmed that the improvements found in double-mutants are not due to the genetic background variation resulting from *mdx*-P2X7^−/−^ breeding, as this would have had an impact in all mouse lines.

### P2RX7 Ablation Reduces the Inflammatory Signature of Dystrophic Muscles

Chronic inflammation is an important pathological feature in DMD and *mdx* [34], and corticosteroids have beneficial, albeit limited, effects in both [35]. Macrophages, along with T-lymphocytes and neutrophils, make up the bulk of immune cell infiltrations, and M1 pro-inflammatory macrophages dominate and add to the damage. However, M2 macrophages are also present and essential for muscle regeneration [36]. Therefore, a non-selective attenuation of immune cell functions carries the risk of interfering with regenerative processes.

**Fig 4:**
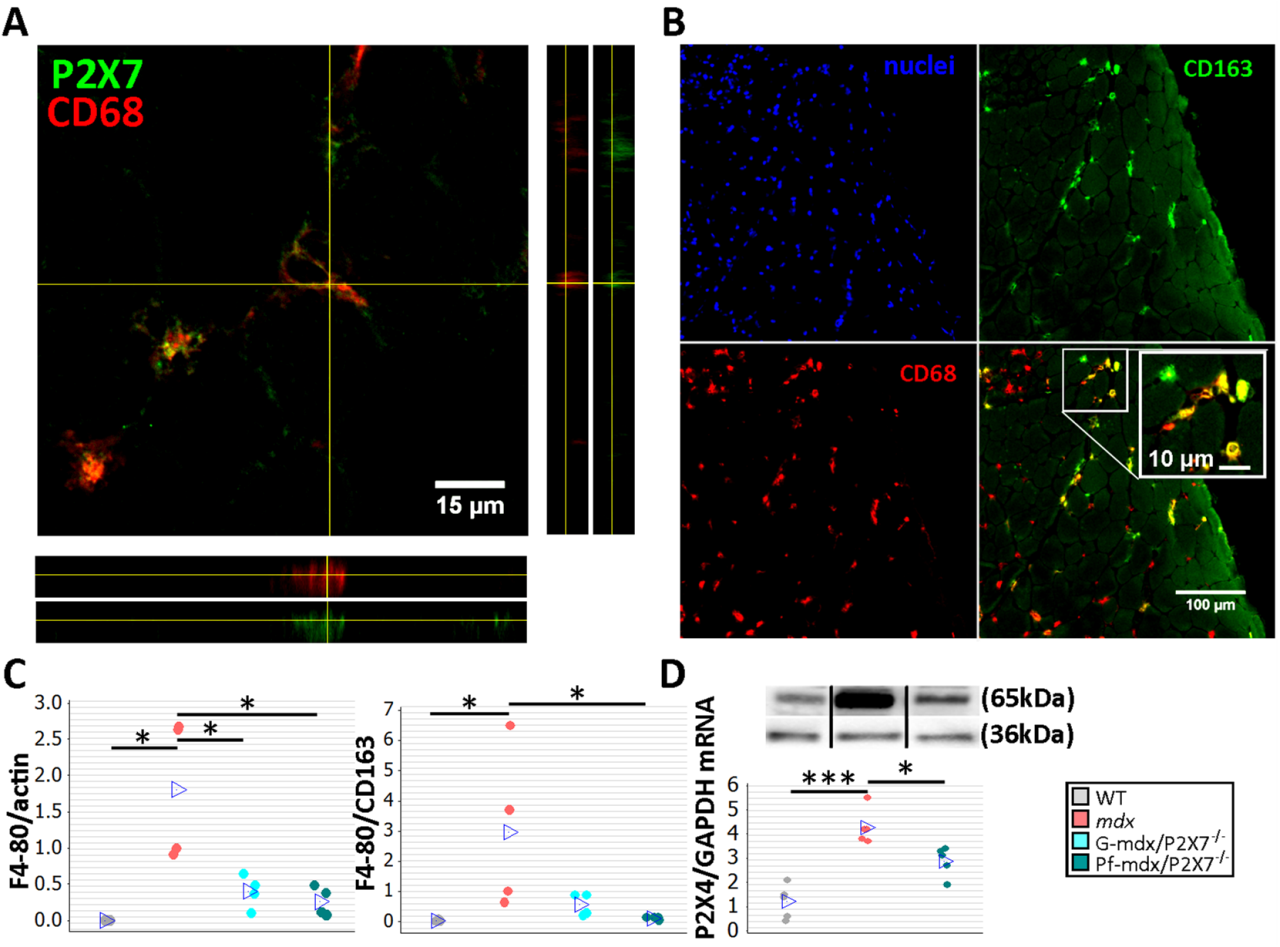
*P2RX7* ablation in *mdx* mice reduces macrophage infiltration. (A) Immunofluorescence co-localization of CD68 macrophage marker with P2RX7. To confirm spatial co-localization, side and bottom panels show overlapping *z*-plane images. (B) M2 macrophage marker (CD163) co-localization with a subset of CD68-positive cells within the inflammatory infiltrate regions in *mdx* muscle. Blue signal identifies cell nuclei. (C) F4/80 macrophage marker levels shown as normalized average Western blot values (left) and F4/80 relative to CD163 (right). Significantly less F4/80 was found in Pf-*mdx*/P2X7^−/−^ and G-*mdx*/P2X7^−/−^ muscles compared to *mdx* muscles (Kruskal-Wallis H test, *H* = 7.73, df = 2, *n* = 4, *p* = 0.021; Mann-Whitney U test, Pf-*mdx*/P2X7^−/−^ versus *mdx*, *W* = 26.0, *n* = 4, *p* = 0.030; G-*mdx*/P2X7^−/−^ versus *mdx*, *W* = 26.0, *n* = 4, *p* = 0.030), while the ratio of F4/80 level to CD163 level, denoting M1/M2 macrophage ratio, was significantly reduced in Pf-*mdx*/P2X7^−/−^ muscles (Kruskal-Wallis H test, *H* = 8.77, df = 2, *n* = 4, *p* = 0.012; Mann-Whitney U test, *W* = 26.0, *n* = 4, *p* = 0.030). (D) A representative Western blot (top) illustrating decreased P2RX4 protein levels, and corresponding qPCR data (bottom) showing decreased expression of P2RX4 mRNA in Pf-*mdx*/P2X7^−/−^ gastrocnemius compared to *mdx* gastrocnemius (ANOVA, *F* = 25.96, df = 2, *n* = 5, *p <* 0.001; Tukey’s test, *mdx* versus C57, *p <* 0.001; Pf-*mdx*/P2X7^−/−^ versus *mdx*, *p* = 0.017; Pf-*mdx*/P2X7^−/−^ versus WT, *p* = 0.005). *p < 0.05, ***p < 0.001. Use of separate Western blots is indicated by solid black lines.

Given P2RX7’s role in induction of inflammation and expression in most immune cells, including in macrophages [14,19], we studied the effects of receptor ablation on the inflammatory signature of dystrophic muscles. To identify major changes, we used inflammatory pathways qPCR panel array analysis (S1 Table) followed by immunodetection. As expected, P2RX7 co-localized with macrophage markers, and the CD163 immunofluorescence associated with the M2 macrophages [36] was present in a subset of CD68-positive cells infiltrating *mdx* muscles (Fig 4A and 4B).

Comparisons of immune cells in *mdx* and *mdx*/P2X7^−/−^ muscles at 4 wk showed that F4/80 pan-macrophage marker levels were lower in both *mdx*/P2X7^−/−^ strains (Fig 4C, left panel; Pf-*mdx*/P2X7^−/−^ versus *mdx*, *p* = 0.030; G-*mdx*/P2X7^−/−^ versus *mdx*, *p* = 0.030), and the F4/80 to CD163 (pro-inflammatory to pro-regenerative) marker ratio was also significantly reduced in Pf-*mdx*/P2X7^−/−^ compared to *mdx* mice (Fig 4C, right panel; *p* = 0.030). P2RX4 expression was significantly lower in Pf-*mdx*/P2X7^−/−^ muscles (Fig 4D; *mdx* versus WT, *p <* 0.001; Pf-*mdx*/P2X7^−/−^ versus *mdx*, *p* = 0.017; Pf-*mdx*/P2X7^−/−^ versus WT, *p* = 0.005), which agreed with the presence of this receptor in infiltrating macrophages [37] and thus further confirmed reduced macrophage loads. This result also excluded potential compensatory overexpression of this receptor in the absence of P2RX7.

**Fig 5:**
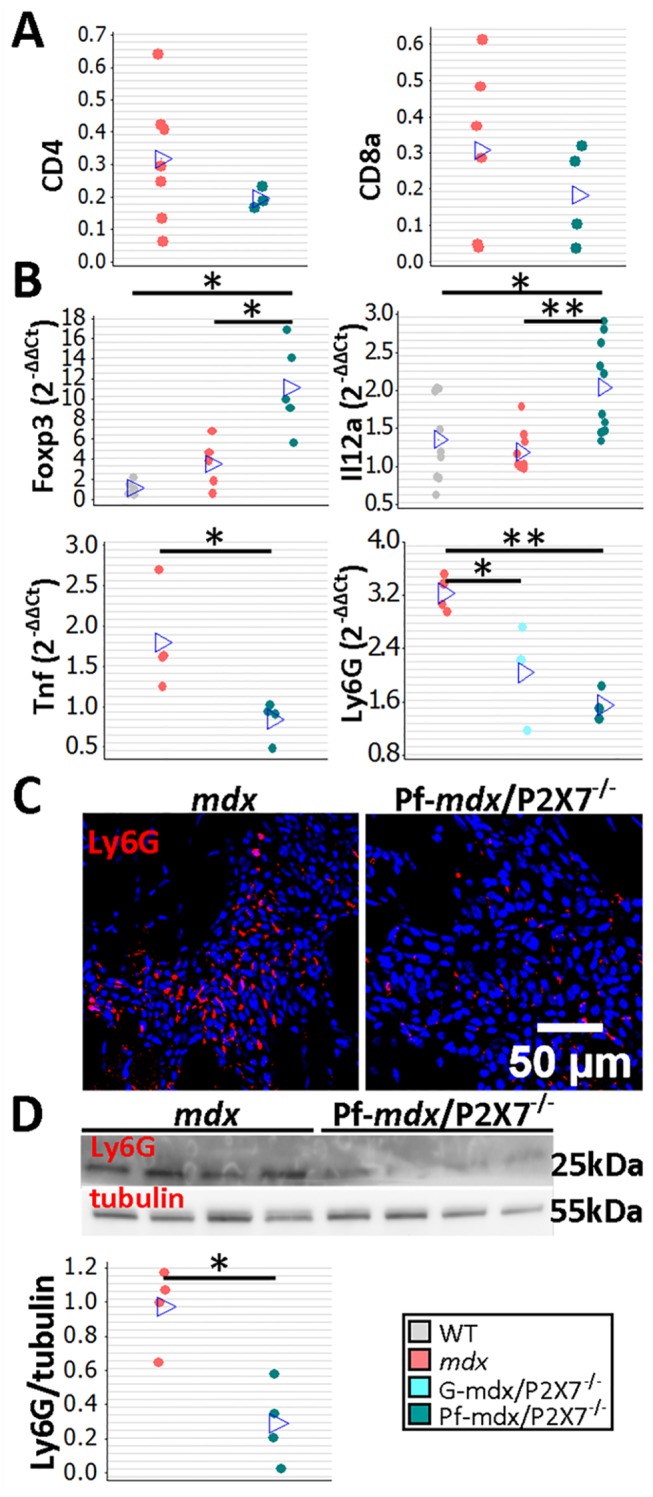
*P2RX7* ablation in *mdx* mice reduces the inflammatory signature. (A) Enumeration of CD4- and CD8-positive cells in TA muscle from *mdx* and Pf-*mdx*/P2X7^−/−^ mice showed no statistical difference in these cell numbers between the two genotypes (CD4 *t*-test, *t* = 0.97, df = 7, *p* = 0.366; CD8 *t*-test, *t* = 0.95, df = 8, *p* = 0.372). This finding corresponded with CD4 and CD8 qPCR data (S1 Table). (B) Selected results of qPCR gene expression analyses using inflammatory panels: relative expression levels (2^−ΔΔCT^) in muscle-derived mRNAs from *mdx* and *mdx*/P2X7^−/−^ mice demonstrate significant differences in expression levels of Foxp3 (Kruskal-Wallis H test, *H* = 10.26, df = 2, *n* = 5, *p* = 0.006; Mann-Whitney U test, Pf-*mdx*/P2X7^−/−^ versus *mdx*, *W* = 16.0, *n* = 5, *p* = 0.022), IL12α (Kruskal-Wallis H test, *H* = 11.08, df = 2, *n* = 9, 10, 10, *p* = 0.004; Mann-Whitney U test, Pf-*mdx*/P2X7^−/−^ versus *mdx*, *W* = 61.0, *n* = 10, *p* = 0.001), TNFα (*t*-test, *t* = 2.86, df = 6, *p* = 0.029), and Ly6G (ANOVA, *F* = 14.68, df = 2, *n* = 3, 4, 4, *p* = 0.002; Tukey’s test, Pf-*mdx*/P2X7^−/−^ versus *mdx*, *p* = 0.002) transcripts in *mdx*/P2X7^−/−^ mice compared to *mdx*. Lower expression of *Ly6G* mRNA corresponded with a lower number of Ly6G immunopositive neutrophils (red signal in Ly6G immunolocalization micrograph) (C) and significantly lower Ly6G protein levels (D) in Pf-*mdx*/P2X7^−/−^ compared to *mdx* muscles (*t*-test, *t* = 4.14, df = 6, *p* = 0.006). Blue signal—Hoechst nuclear counterstaining. *p < 0.05, **p < 0.005.

The CD4 and CD8 cell numbers were not significantly affected by *P2RX7* ablation (Fig 5A; *p >* 0.05), but a significant increase in Foxp3 and IL12α expression in *mdx*/P2X7^−/−^ muscles (Fig 5B; Pf-*mdx*/P2X7^−/−^ versus *mdx*: Foxp3, *p* = 0.022, and IL12α, *p* = 0.001; see also S1 Table) indicated a shift towards T_reg_ cells, known to suppress immune responses [38]. This result showed that P2RX7 depletion ameliorates tissue inflammation by promoting T_reg_ cell functions [39] also in dystrophic muscles.

Together, these data indicate a significantly reduced inflammatory profile in 4-wk-old Pf-*mdx*/P2X7^−/−^ muscles. Moreover, the decreased TNFα expression (Fig 5B; *p* = 0.029; S1 Table) agreed with its production being dependent on P2RX7 function [19] but was also an important result given that pharmacological interference with this inflammatory mediator has been shown to reduce DMD pathogenesis [40]. The significantly reduced neutrophil marker Ly6G mRNA expression found in Pf-*mdx*/P2X7^−/−^ muscles (Fig 5B; Pf-*mdx*/P2X7^−/−^ versus *mdx*, *p* = 0.002; S1 Table) was confirmed by both immunolocalization (Fig 5C) and Western blotting (Fig 5D; *p* = 0.006). Clearly, in agreement with recent studies [41], P2RX7 ablation has a widespread impact on inflammatory cell migration, and this correlates with significant improvements in dystrophic muscles.

### Dystrophic Muscles Present an Early Fibrotic Signature, Which Is Reduced by *P2RX7* Ablation

**Fig 6:**
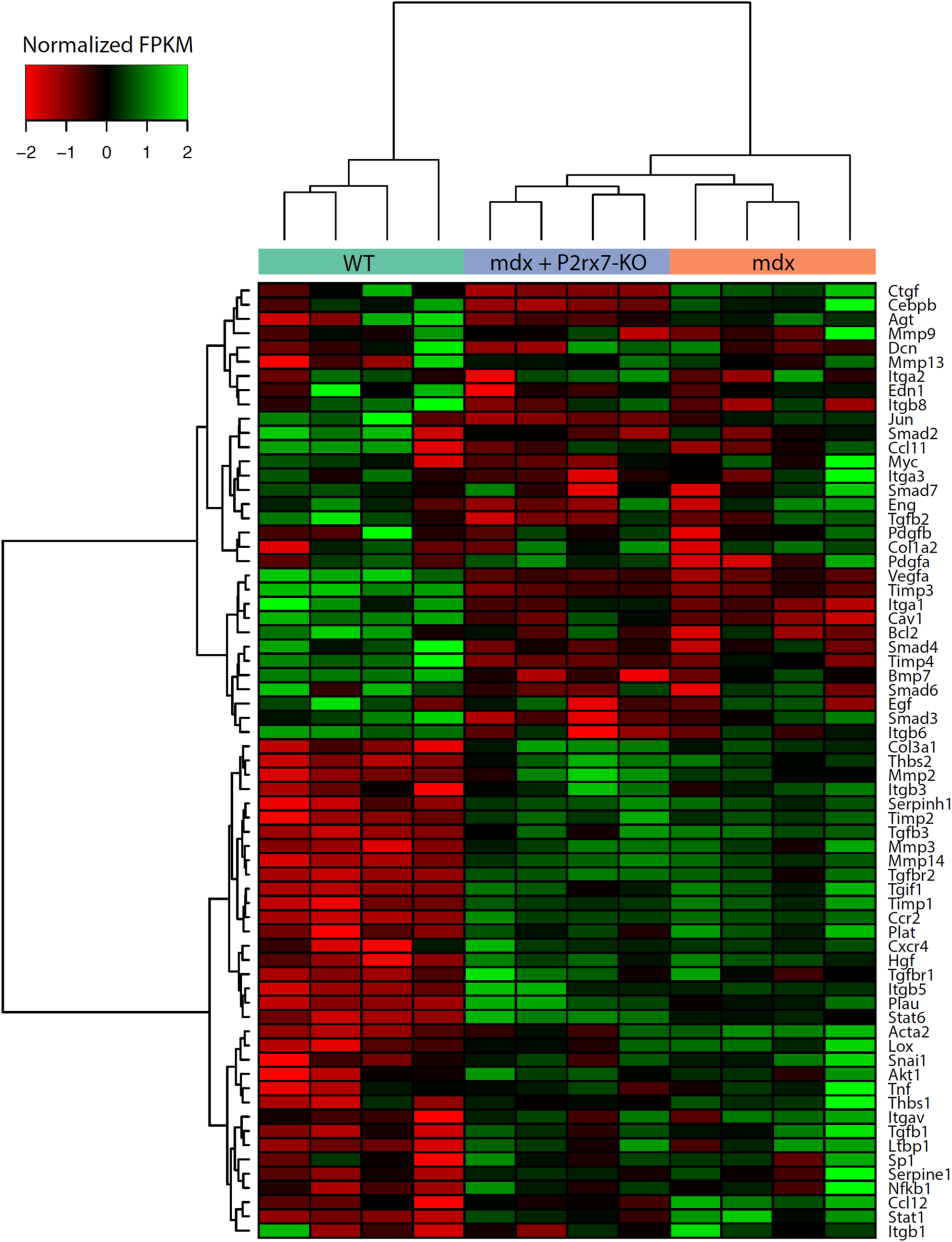
Expression of genes associated with fibrosis as measured by RNA-Seq. Fragments per kilobase per million fragments mapped (FPKM) values for each sample were obtained using Cuffnorm (Tuxedo suite), log-transformed, and normalized to zero mean and unit standard deviation (rows with missing expression values were removed). Hierarchical clustering was performed using the Ward’s method [42]. KO, knockout.

Unlike in DMD, the *mdx* mouse shows little fibrosis in limb muscles. Surprisingly, combined RNA-Seq and qPCR analyses of 4-wk-old leg muscle samples revealed a clear pro-fibrotic gene expression signature at this early disease stage (Fig 6).

The expression pattern of genes involved in the regulation of fibrosis showed significant up-regulation of pro-fibrotic and down-regulation of anti-fibrotic genes (S2 Table; S2 Fig). Importantly, *P2RX7* ablation reduced this early fibrotic signature (Figs 6 and S2), with many genes expressed at levels resembling those in WT muscles (for specific *p-*values see S2 Table). This gene-level expression analysis not only uncovered an early, pro-fibrotic environment in *mdx* muscles but also confirmed that the widespread effects of *P2RX7* ablation include lessening of this phenotype, which is one of the critically important abnormalities of the dystrophic pathology.

### *P2RX7* Ablation Produces Long-Term Improvements in Skeletal and Cardiac Muscles

**Fig 7:**
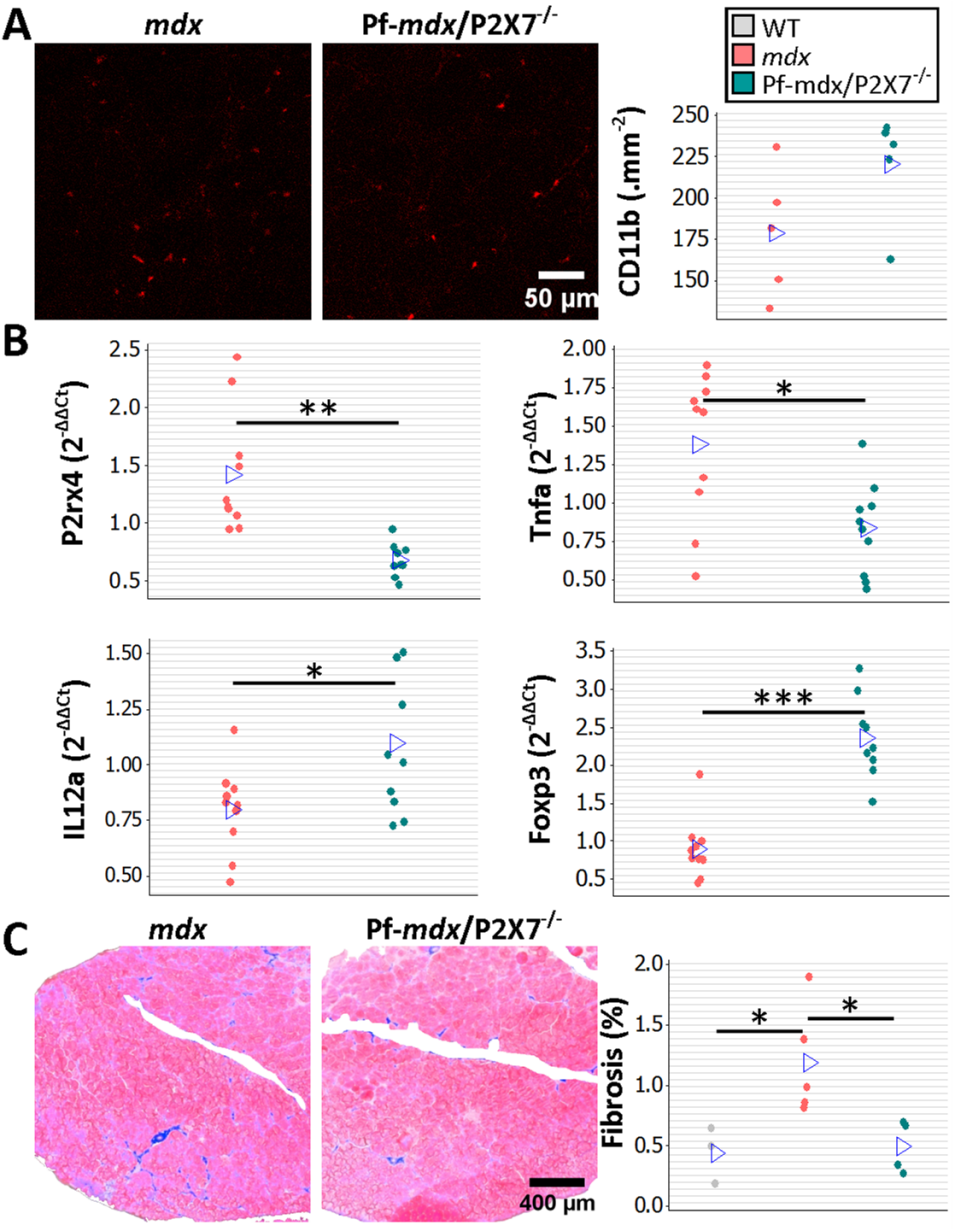
*P2RX7* ablation reduces inflammation and fibrosis in 20-mo-old tibialis anterior muscles. (A) Representative immunofluorescence micrographs (left) and enumeration of CD11b-expressing cells in 20-mo-old TA showing no significant difference in the numbers of infiltrating leukocytes in Pf-*mdx*/P2X7^−/−^ compared to *mdx* muscles (*t*-test, *t* = 1.82, df = 8, *p* = 0.107). (B) qPCR gene expression analyses: relative expression levels (2^−ΔΔCT^) in muscle-derived mRNAs from *mdx* and *mdx*/P2X7^−/−^ TA demonstrate significant decreases in P2RX4 (*t*-test, *t* = 4.04, df = 17, *p* = 0.001) and TNFα (*t*-test, *t* = 3.07, df = 18, *p* = 0.006), with concomitant increases in expression levels of IL12α (*t*-test, *t* = 2.56, df = 18, *p* = 0.020) and Foxp3 (*t*-test, *t* = 6.8, df = 17, *p <* 0.001) in Pf-*mdx*/P2X7^−/−^ compared to *mdx* muscles. (C) Representative images of trichrome staining (left) and trichrome average intensities in 20-mo-old TA muscles demonstrating a significant decrease in fibrosis in Pf-*mdx*/P2X7^−/−^ compared to *mdx* mice (ANOVA, *F* = 6.18, df = 2, *n* = 3, 5, 4, *p* = 0.020; Tukey’s test, Pf-*mdx*/P2X7^−/−^ versus *mdx*, *p* = 0.038). *p < 0.05, **p < 0.005, ***p < 0.001.

To assess the long-term effects of *P2RX7* ablation, we tested 20-mo-old limb, diaphragm, and cardiac muscles [21]. As expected at this stage, the leukocyte (CD11b-positive cell) numbers were low and were not different between Pf-*mdx*/P2X7^−/−^ and *mdx* TA muscles (Fig 7A; *p >* 0.05).

**Fig 8:**
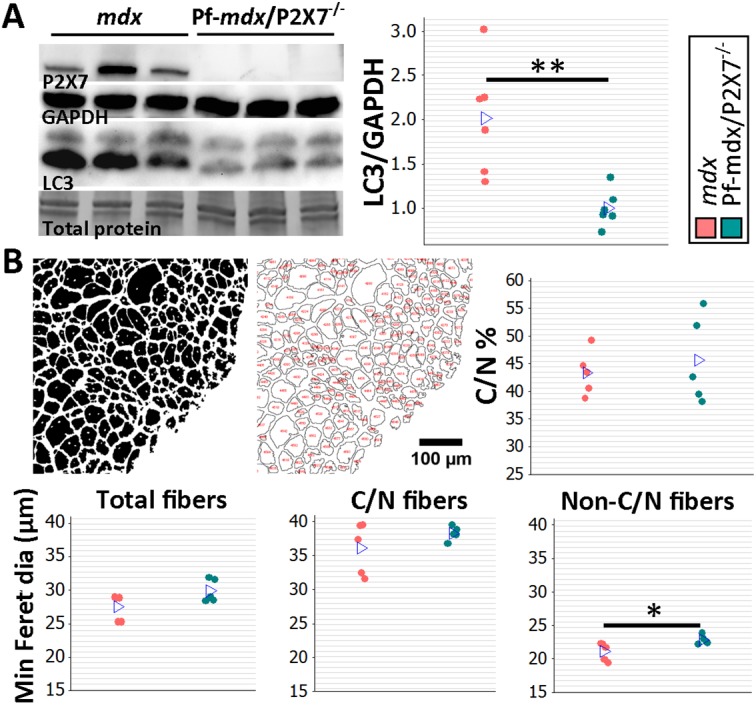
*P2RX7* ablation reduces the pathology in 20-mo-old tibialis anterior muscles. (A) Representative Western blots (left) confirming P2RX7 protein expression in 20-mo-old TA and illustrating decreased autophagy (LC3I to LC3II shift), with the results in the graph showing lower levels of LC3II relative to GAPDH in TA from 20-mo-old Pf-*mdx*/P2X7^−/−^ compared to *mdx* mice (*t*-test, *t* = 3.74, df = 10, *p* = 0.004). Total protein staining is shown to illustrate the equal protein loading. (B) Representative ImageJ output masks from morphometric analyses of TA fibers (left) and graphs showing representative results (right and bottom). While no differences were found in the proportion of C/N fibers or the average minimum Feret diameter of total or C/N fibers (*t*-test, *t* = 0.57, df = 8, *p* = 0.586; *t* = 2.04, df = 8, *p* = 0.076; *t* = 1.25, df = 8, *p* = 0.248, respectively), there was a significant increase in the minimum Feret diameter of non-C/N fibers in *mdx*/P2X7^−/−^ compared to *mdx* muscles (*t*-test, *t* = 2.58, df = 8, *p* = 0.032). *p < 0.05, **p < 0.005.

However, qPCR analyses showed significantly lower expression of P2RX4 (*p* = 0.001) and TNFα (*p* = 0.007) genes and significantly increased Foxp3 (*p <* 0.001) and IL12α (*p* = 0.020) expression levels in *mdx*/P2X7^−/−^ TA samples (Fig 7B). These findings agreed with the results in 4-wk-old samples and demonstrated that *P2RX7* ablation has a long-term anti-inflammatory effect in dystrophic muscles. Moreover, the statistically significant reduction in trichrome staining in 20-mo-old Pf-*mdx*/P2X7^−/−^ TA (Fig 7C; Pf-*mdx*/P2X7^−/−^ versus *mdx*, *p* = 0.038) confirmed reduced muscle fibrosis across ages. P2RX7 expression was detectable in *mdx* muscles at this age (Fig 8A), and the reduced autophagy in 20-mo-old *mdx*/P2X7^−/−^ muscles (Fig 8A; *p* = 0.004) corresponded with the results obtained in 4-wk-old samples and extended our earlier observation of the protective effect of *P2RX7* ablation against ATP-induced autophagic muscle death [12].

Finally, there was no difference in the percentage of C/N fibers (*p >* 0.05), and measurements of the average minimum Feret diameter showed no differences in total (*p >* 0.05) and C/N (*p >* 0.05) fibers, while there was a significant increase in Feret diameter (*p* = 0.032) in non-C/N fibers from 20-mo-old Pf-*mdx*/P2X7^−/−^ TA (Fig 8B).

**Fig 9:**
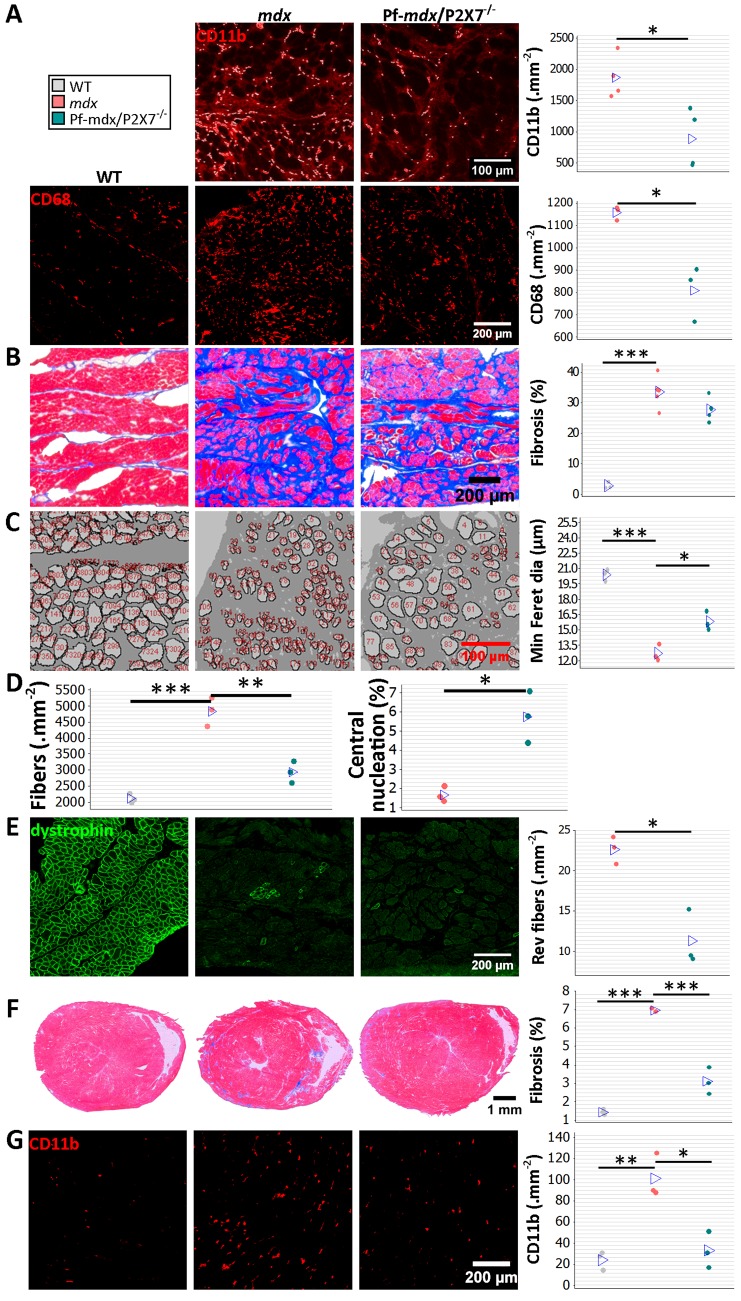
*P2RX7* ablation continues to reduce dystrophic pathology in 20-mo-old diaphragm and heart. (A) Representative immunofluorescence micrographs (left) and enumeration of CD11b- and CD68-expressing cells in 20-mo-old diaphragms showing reduced numbers of infiltrating leukocytes (CD11b *t-*test, *t* = 3.68, df = 6, *p* = 0.015) and macrophages (CD68^+^ *t*-test, *t* = 4.73, df = 4, *p* = 0.009) in Pf-*mdx*/P2X7^−/−^ compared to *mdx* diaphragms. (B) Trichrome staining (left) and its average intensity in 20-mo-old diaphragms demonstrating no increase in fibrosis in Pf-*mdx*/P2X7^−/−^ over *mdx* mice (ANOVA, *F* = 60.32, df = 2, *n* = 5, 5, 3, *p <* 0.001; Tukey’s test, Pf-*mdx*/P2X7^−/−^ versus *mdx*, *p* = 0.095; permutation analysis, Pf-*mdx*/P2X7^−/−^ versus *mdx*, *F* = 4.47, *p* = 0.095). (C) Representative ImageJ output masks from morphometric analyses of diaphragm fibers (left) demonstrating the increased average minimum Feret diameter in *mdx*/P2X7^−/−^ compared to *mdx* diaphragms (ANOVA, *F* = 75.17, df = 2, *n* = 3, *p <* 0.001; Tukey’s test, Pf-*mdx*/P2X7^−/−^ versus *mdx*, *p* = 0.006; permutation analysis, Pf-*mdx*/P2X7^−/−^ versus *mdx*, *F* = 20.01, *p* = 0.099). (D) Graphs showing a lower total number of diaphragm fibers per unit area in Pf-*mdx*/P2X7^−/−^ versus *mdx* mice (left; ANOVA, *F* = 52.77, df = 2, *n* = 3, *p <* 0.001; Tukey’s test, Pf-*mdx*/P2X7^−/−^ versus *mdx*, *p* = 0.001; Pf-*mdx*/P2X7^−/−^ versus WT, *p* = 0.051) and the increased proportion of C/N fibers (right; *t-*test, *t* = 5, df = 4, *p* = 0.008). (E) Dystrophin immunofluorescence in representative transverse sections of 20-mo-old diaphragms showing the typical staining (green signal) in dystrophin-positive muscles and clusters of revertant dystrophin-positive fibers in dystrophic samples. The data show significantly fewer revertant fibers in 20-mo-old Pf-*mdx*/P2X7^−/−^ than in *mdx* diaphragms (*t-*test, *t* = 5.12, df = 4, *p* = 0.007). (F) Representative trichrome staining (left) of whole hearts from 20-mo-old mice showing a significant decrease in cardiac muscle damage (histological lesions) and average area of fibrosis (blue signal in trichrome staining) in Pf-*mdx*/P2X7^−/−^ versus *mdx* mice (ANOVA, *F* = 166.29, df = 2, *n* = 4, 3, 3, *p <* 0.001; Tukey’s test, Pf-*mdx*/P2X7^−/−^ versus *mdx*, *p <* 0.001). (G) Representative examples of CD11b^+^ leukocyte marker staining (left; red immunofluorescence) and infiltrating cell counts demonstrating fewer infiltrations in Pf-*mdx*/P2X7^−/−^ compared to *mdx* hearts (ANOVA, *F* = 19.65, df = 2, *n* = 3, *p* = 0.002; Tukey’s test, Pf-*mdx*/P2X7^−/−^ versus *mdx*, *p* = 0.005). *p < 0.05, **p < 0.005, ***p < 0.001.

Notably, diaphragm is the *mdx* muscle that shows mild pathology at early stages and undergoes progressive degeneration that becomes prominent from week 15 [21]. The numbers of CD11b^+^ and CD68^+^ inflammatory cells were significantly lower in 20-mo-old Pf-*mdx*/P2X7^−/−^ diaphragms (Fig 9A; CD11b^+^, *p* = 0.015; CD68^+^, *p* = 0.009), confirming that the long-term reduction of the pro-inflammatory phenotype also occurs in this progressively damaged muscle.

Similar levels of connective tissue in trichrome staining (Fig 9B; Pf-*mdx*/P2X7^−/−^ versus *mdx*, *p* = 0.095) showed that the altered profile of cells infiltrating double-mutant muscles did not alter diaphragm fibrosis [36]. There was, however, a statistically significant increase in muscle fiber Feret diameter (Fig 9C; Pf-*mdx*/P2X7^−/−^ versus *mdx*, *p* = 0.006), concomitant with a lower total number of diaphragm fibers per unit area (Fig 9D; Pf-*mdx*/P2X7^−/−^ versus *mdx*, *p* = 0.001; Pf-*mdx*/P2X7^−/−^ versus WT, *p* = 0.051), while the percentage of C/N fibers was lower in *mdx* than in Pf-*mdx*/P2X7^−/−^ diaphragms (Fig 9D, *p* = 0.008). Revertant fibers were present in a lower number in Pf-*mdx*/P2X7^−/−^ than *mdx* diaphragms (Fig 9E; *p* = 0.007). Dystrophin-expressing revertant fibers arise due to spontaneous exon skipping. These rare events take place only in proliferating precursor cells, which are activated by muscle degeneration/regeneration. Therefore, in experimental paradigms not affecting splicing of the dystrophin transcript, fewer revertant fibers have been used as an indicator that fewer degeneration/regeneration cycles occurred over the muscle lifetime [43,44].

DMD patients who survive to the third decade present with cardiomyopathy, and heart failure becomes a common cause of death [22]. We analyzed hearts from 20-mo-old mice and found significantly decreased cardiac muscle fibrosis (Pf-*mdx*/P2X7^−/−^ versus *mdx*, *p <* 0.001), measured as area occupied by connective tissue in trichrome staining and structural damage (Fig 9F), coinciding with fewer CD11b-positive leukocytes infiltrating Pf-*mdx*/P2X7^−/−^ compared to *mdx* hearts (Fig 9G; Pf-*mdx*/P2X7^−/−^ versus *mdx*, *p* = 0.005).

### *P2RX7* Ablation Improves *mdx* Muscle Strength and Endurance In Vivo and Reduces Cognitive and Behavioral Abnormalities

**Fig 10:**
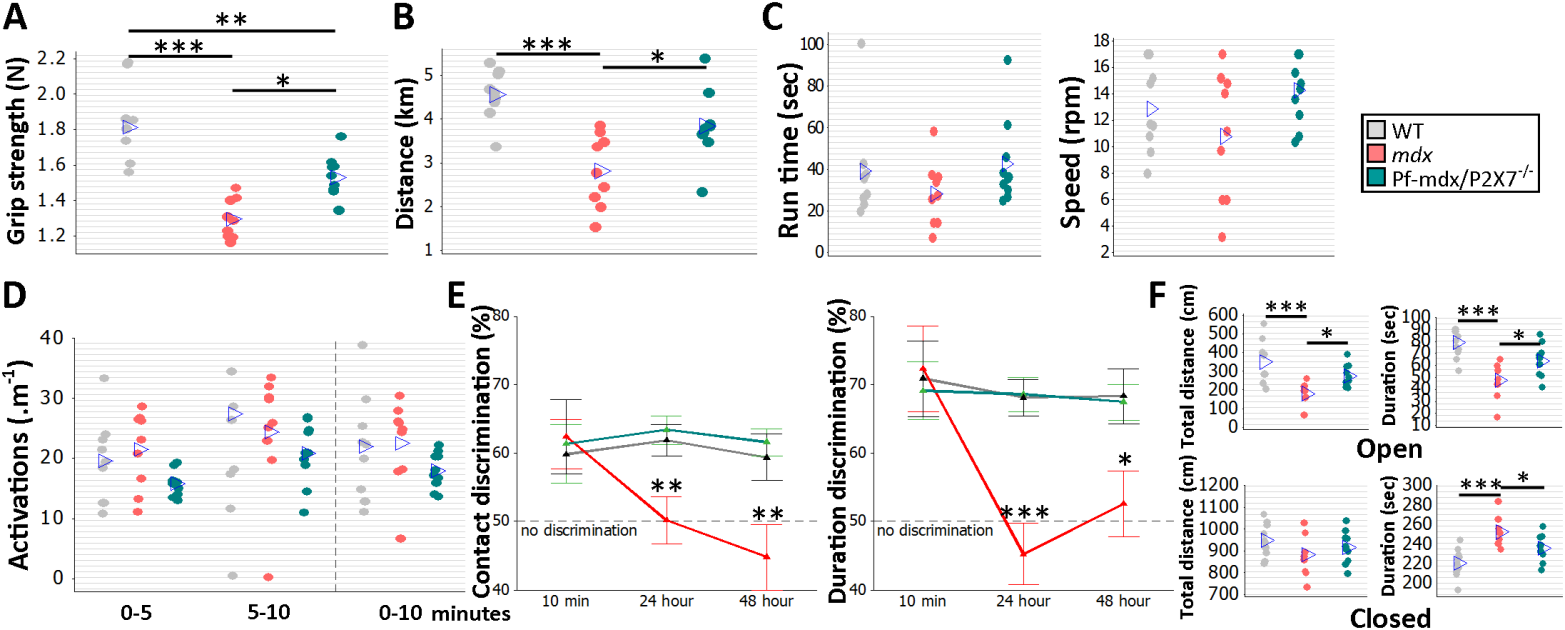
*P2RX7* ablation improves *mdx* muscle strength and endurance and object recognition memory and decreases anxiety in vivo. Forelimb grip strength (A) and voluntary wheel run distance (B) were significantly greater in Pf-*mdx*/P2X7^−/−^ compared to *mdx* mice (grip strength ANOVA, *F* = 22.99, df = 2, *n* = 9, 9, 10, *p <* 0.001; Tukey’s test, Pf-*mdx*/P2X7^−/−^ versus *mdx*, *p* = 0.0118; run distance ANOVA, *F* = 12.73, df = 2, *n* = 9, 9, 10, *p <* 0.001; Tukey’s test, Pf-*mdx*/P2X7^−/−^ versus *mdx*, *p* = 0.016). The rotarod test (C) showed no difference for total average run time and average speed (run time ANOVA, *F* = 1.23, df = 2, *n* = 9, 9, 10, *p* = 0.310; Tukey’s test, Pf-*mdx*/P2X7^−/−^ versus *mdx*, *p* = 0.300; speed ANOVA, *F* = 2.23, df = 2, *n* = 9, 9, 10, *p* = 0.129; Tukey’s test, Pf-*mdx*/P2X7^−/−^ versus *mdx*, *p* = 0.109), and the parallel rod floor test (D) showed no significant differences in the average number of activations over several run time-spans between WT, *mdx*, and Pf-*mdx*/P2X7^−/−^ mice (ANOVA, *F* = 2.6, 0.62, 1.34; df = 2; *n* = 9, 9, 10; *p* = 0.094, 0.545, 0.212; at 0–5, 5–10, and 0–10 min, respectively). In the object recognition test (E) there was no significant difference between genotypes at 10-min retention delay, but at 24 h and 48 h, both duration and contact discrimination were significantly different from the 50% chance level for the Pf-*mdx*/P2X7^−/−^ mice, but not for *mdx* mice. Memory retention in Pf-*mdx*/P2X7^−/−^ mice was equal to that in WT mice, while *mdx* mice performed at a lower level than WT (contact discrimination 10 min, 24 h, 48 h ANOVA; *F* = 0.08, 7.37, 6.83; df = 2; *n* = 10, 9, 10; *p* = 0.922, 0.003, 0.004; Tukey’s test, Pf-*mdx*/P2X7^−/−^ versus *mdx*, *p* = 0.984, 0.004, 0.006; Pf-*mdx*/P2X7^−/−^ versus WT, *p* = 0.970, 0.908, 0.890; *mdx* versus WT, *p* = 0.916, 0.012, 0.017; duration discrimination 10 min, 24 h, 48 h ANOVA; *F* = 0.09, 16.17, 5.1; df = 2; *n* = 10, 9, 10; *p* = 0.913, < 0.001, = 0.013; Tukey’s test, Pf-*mdx*/P2X7^−/−^ versus *mdx*, *p* = 0.905, < 0.001, = 0.031; Pf-*mdx*/P2X7^−/−^ versus WT, *p* = 0.969, 0.994, 0.985; *mdx* versus WT, *p* = 0.980, < 0.001, = 0.021,). (F) In the elevated zero maze anxiety test, both the duration and the distance travelled by Pf-*mdx*/P2X7^−/−^ mice within the open arm of the maze were greater than those of *mdx* mice, and *mdx* mice performed at a lower level than WT (distance and duration ANOVA, *F* = 10.51, 11.76; df = 2; *n* = 10, 9, 10; *p <* 0.001, < 0.001; Tukey’s test, Pf-*mdx*/P2X7^−/−^ versus *mdx*, *p* = 0.045, 0.047; Pf-*mdx*/P2X7^−/−^ versus WT, *p* = 0.109, 0.060; *mdx* versus WT, *p* < 0.001, < 0.001). In the closed arm (F, bottom), there was no difference in distance travelled, but Pf-*mdx*/P2X7^−/−^ mice spent less time in this arm than *mdx* mice, and *mdx* mice more time than WT (distance and duration ANOVA, *F* = 1.42, 11.76; df = 2; *n* = 10, 9, 10; *p* = 0.259, < 0.001; Tukey’s test, Pf-*mdx*/P2X7^−/−^ versus *mdx*, *p* = 0.687, 0.047; Pf-*mdx*/P2X7^−/−^ versus WT, *p* = 0.659, 0.060; *mdx* versus WT, *p* = 0.230, < 0.001). *p < 0.05, **p < 0.005, ***p < 0.001.

To analyze the effects of receptor ablation on muscle function in vivo, groups of 8- to 12-wk-old WT, *mdx*, and Pf-*mdx*/P2X7^−/−^ male mice followed a functional test regime consisting of forelimb grip strength, rotarod, voluntary wheel activity, and parallel rod floor running. Significant improvements in grip strength (Fig 10A; *p* = 0.012) and muscle endurance (Fig 10B; *p* = 0.016) were observed in *mdx*/P2X7^−/−^ compared to *mdx* mice. A lack of changes in latency time to fall off the rotarod and in performance in the parallel rod floor tests (Fig 10C, run time and speed; Fig 10D, activations; all *p* > 0.05 for *mdx*/P2X7^−/−^ versus *mdx*) excluded differences in motor coordination between these strains.

Cognitive and behavioral impairment is a well-defined feature of DMD: the overall IQ of DMD patients is one standard deviation below the mean of the unaffected population [2]. Distinct dystrophin isoforms are expressed in neurons and glia [45], and P2RX7 is recognized as a gatekeeper of inflammatory processes between these two cell types [46]. Treatments targeting the adult *mdx* brain produced some improvement [47], proving that at least some abnormalities are reversible. WT, *mdx*, and Pf-*mdx*/P2X7^−/−^ mice were therefore compared in a series of behavioral tests to identify the effect(s) of receptor ablation. Consistent with previous studies [48], *mdx* mice showed impaired long-term recognition memory and enhanced emotional reactivity (Fig 10E; contact discrimination 10 min, 24 h, 48 h; *p* = 0.916, 0.012, 0.017; duration discrimination 10 min, 24 h, 48 h; *p* = 0.980, < 0.001, = 0.021; Fig 10F; open-arm distance and duration; *p* < 0.001, < 0.001; closed-arm distance and duration, *p* = 0.230, < 0.001). Importantly, ablation of P2RX7 in *mdx* mice resulted in a significant improvement in performance in the novel object recognition memory test (Fig 10E; contact discrimination 10 min, 24 h, 48 h Tukey’s test; Pf-*mdx/*P2X7^−/−^ versus *mdx*, *p* = 0.984, 0.004, 0.006; Pf-*mdx/*P2X7^−/−^ versus WT, *p* = 0.970, 0.908, 0.890; duration discrimination Tukey’s test 10 min, 24 h, 48 h; Pf-*mdx*/P2X7^−/−^ versus *mdx*, *p* = 0.905, < 0.001, = 0.031; Pf-*mdx/*P2X7^−/−^ versus WT, *p* = 0.969, 0.994, 0.985) and in reduced anxiety as measured in the elevated zero maze test (Fig 10F; open-arm distance and duration Tukey’s test; Pf-*mdx*/P2X7^−/−^ versus *mdx*, *p* = 0.0450, 0.047; Pf-*mdx*/P2X7^−/−^ versus WT, *p* = 0.109, 0.060; closed-arm distance and duration Tukey’s test; Pf-*mdx*/P2X7^−/−^ versus *mdx*, *p* = 0.687, 0.047; Pf-*mdx*/P2X7^−/−^ versus WT, *p* = 0.659, 0.060). These tests are not affected by motor activity or muscle strength and therefore reflect the positive impact of P2RX7 ablation on the dystrophic central nervous system (CNS).

### *P2RX7* Ablation Reduces Bone Loss in Dystrophic Mice

**Fig 11:**
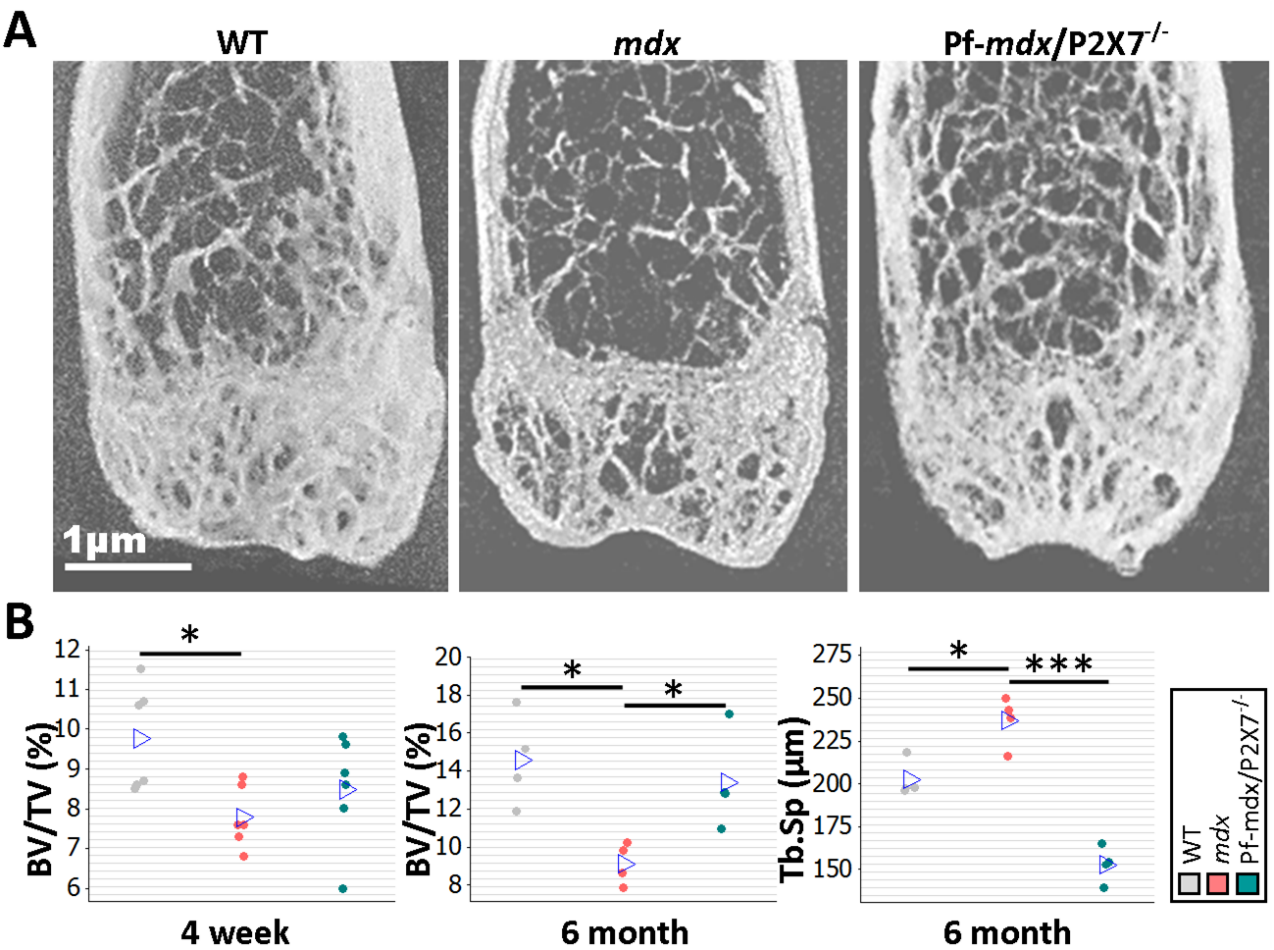
*P2RX7* ablation reduces *mdx* bone loss. (A) Representative μ-CT images of distal femurs from 6-mo-old mice. (B) μ-CT morphometry in 4-wk-old proximal tibiae and 6-mo-old femurs. As early as age 4 wk, the BV/TV ratio in *mdx* mice was reduced, and it remained altered at 6 mo. But P2RX7 ablation significantly improved BV/TV and Tb.Sp parameters in older *mdx*/P2X7^−/−^ mice compared to older *mdx* mice (4-wk BV/TV ANOVA, *F* = 4.29, df = 2, *n* = 6, *p* = 0.034; Tukey’s test, *mdx* versus WT, *p* = 0.029; 6-mo BV/TV ANOVA, *F* = 7.29, df = 2, *n* = 4, *p* = 0.013; Tukey’s test, *mdx* versus WT, *p* = 0.014; Pf-*mdx*/P2X7^−/−^ versus *mdx*, *p* = 0.046; 6-mo Tb.Sp ANOVA, *F* = 48.69, df = 2, *n* = 4, *p <* 0.001; Tukey’s test, *mdx* versus WT, *p* = 0.008; Pf-*mdx*/P2X7^−/−^ versus *mdx*, *p <* 0.001.). *p < 0.05, ***p < 0.001.

Bone structure abnormalities in DMD patients and *mdx* mice were previously believed to have musculoskeletal origins. However, rather than being an effect of asymmetric loss of muscle force, these have recently been confirmed as an independent disease manifestation [3]. μCT bone morphometry analyses (Figs 11A and S3) in WT, *mdx*, and *mdx*/P2X7^−/−^ mice revealed reduced bone mass in *mdx* mice as young as 4 wk, i.e., before any muscle loss that could cause bone structure alterations, and confirmed significant bone abnormalities at 6 mo (Fig 11B; 4-wk BV/TV, *p* = 0.029; 6-mo BV/TV, *p* = 0.014; 6-mo Tb.Sp, *p* = 0.008).

In contrast, in 6-mo-old Pf-*mdx*/P2X7^−/−^ mice, we found significant improvements in BV/TV (*p* = 0.046) and in Tb.Sp (*p <* 0.001) compared to *mdx* mice (Fig 11B). These data demonstrate that *P2RX7* ablation improves the dystrophic bone phenotype.

### P2RX7 Antagonist Treatment Reduces Dystrophic Pathology

**Fig 12:**
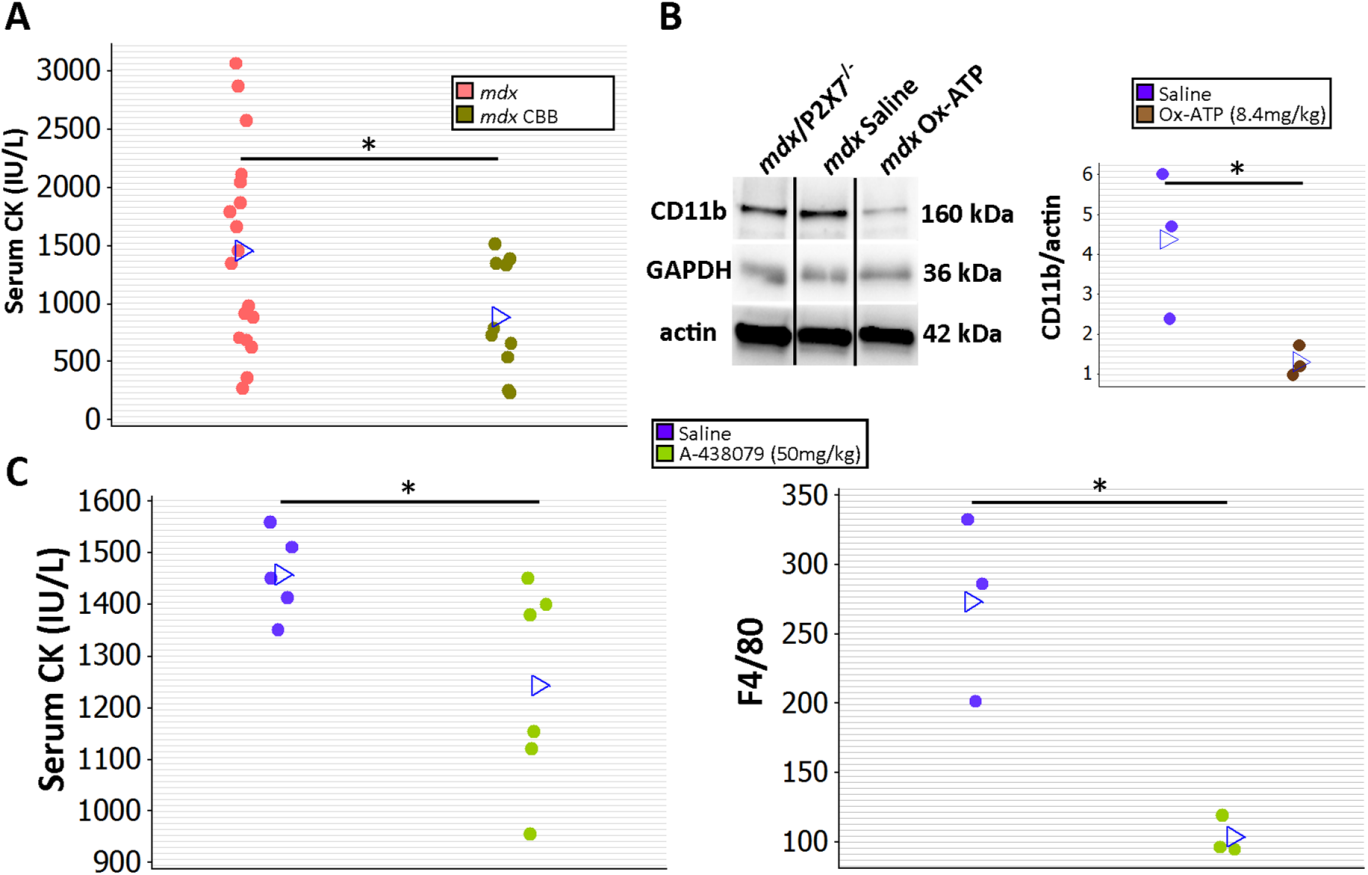
Short-term P2RX7 antagonist administration reduces severity of muscle pathology. (A) Comparison of serum CK levels in *mdx* mice (control) and *mdx* mice injected with the P2RX7 antagonist CBB. Note the high variability of CK levels in the dystrophic sera. Daily administration of CBB over the 4-wk period reduced CK levels (*t-*test, *t* = 2.3, df = 26, *p* = 0.030), in line with the effects of P2RX7 ablation in 4-wk-old Pf-*mdx*/P2X7^−/−^ mice. (B) Representative Western blots (left) and average value plots (right) demonstrating significantly decreased levels of the CD11b leukocyte marker in gastrocnemius muscles of *mdx* mice injected with ox-ATP compared to *mdx* saline-injected controls (*t-*test, *t* = 2.84, df = 4, *p* = 0.047). Use of separate Western blots is indicated by solid black lines. (C) Comparisons of serum CK levels (left) and F4/80^+^ macrophage loads in *mdx* mice showing significant decreases (CK *t-*test, *t* = 2.26, df = 9, *p* = 0.050; F4/80 *t-*test, *t* = 4.34, df = 4, *n* = 3, *p* = 0.012) following 14 daily administrations of the competitive P2RX7 antagonist A-438079. *p < 0.05.

In a preliminary study to determine whether treatment with P2RX7 antagonists can ameliorate dystrophic symptoms in vivo, 2-wk-old *mdx* mice were injected intraperitoneally with CBB or ox-ATP for 4 wk. CBB treatment reduced CK levels (Fig 12A; *p* = 0.030), which correlates with *mdx*/P2X7^−/−^ results presented here and also with previous data showing a reduced number of degeneration/regeneration cycles following CBB administration in 6-mo-old *mdx* mice [9].

Moreover, ox-ATP treatment decreased muscle expression of CD11b, denoting reduced inflammatory cell infiltrations (Fig 12B; *p* = 0.047). Finally, daily administration of 50 mg/kg of the more selective, competitive P2RX7 antagonist A-438079 for only 2 wk [49] decreased CK levels (*p* = 0.050) and significantly reduced the number of infiltrating (F4/80 positive) macrophages (Fig 12C; *p* = 0.012), again in agreement with improvements found in *mdx*/P2X7^−/−^.

## Discussion

**Fig 13:**
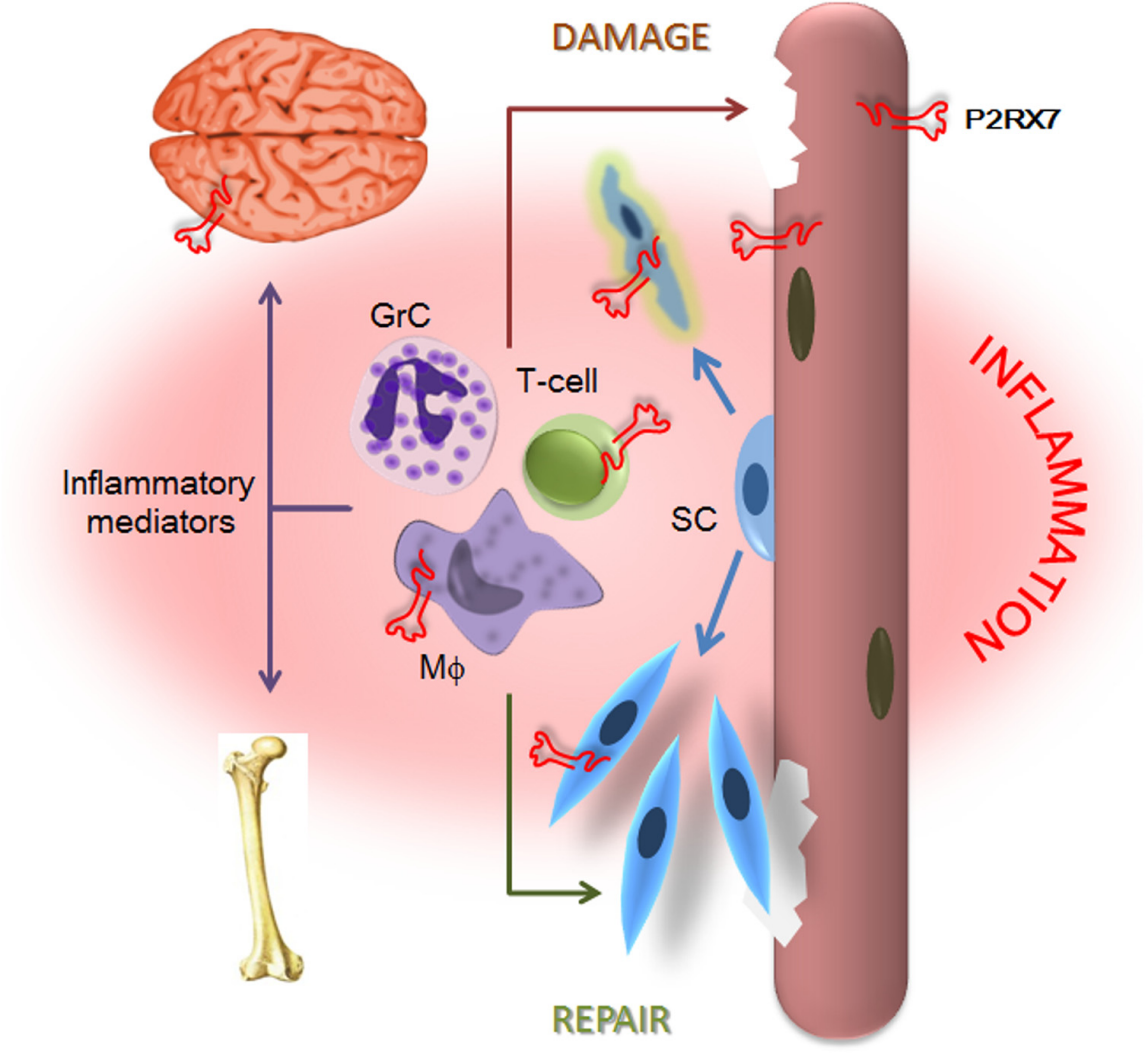
P2X7 purinoceptor involvement in the dystrophic pathology. Absence of dystrophin and resulting loss of the DAP complex lead to myofiber damage. Degenerating/dying muscle releases large quantities of DAMPs, including ATP, which trigger chronic inflammation. P2RX7 activation on dystrophic myofibers exacerbates injury by promoting intracellular Ca^2+^ build-up and autophagic cell death. Infiltrating macrophages (Mφ), T-cells, and granulocytes (GrC) cause further myofiber damage, while chronically elevated levels of inflammatory mediators disturb normal brain and bone functions. Chronic inflammation also reduces repair by altering satellite cell (SC) activation and muscle precursor cell differentiation, while high eATP levels combined with P2RX7 overexpression contribute to their death and thus reduce muscle regeneration further still.

The data we present here demonstrate that *P2RX7* ablation in the most widely used animal model of DMD produced significant improvements in key functional and molecular disease parameters in dystrophic leg muscles at 4 wk and in legs, diaphragms, and hearts at 20 mo, i.e., at all the stages where the model reproduces the DMD pathology [21]. In addition to the alleviation of muscle disease, decreased inflammation and reduced non-muscle symptoms in CNS and bone were also clearly evident. This wide therapeutic impact reflects convergence of *P2RX7* ablation on multiple pathological mechanisms (summarized in Fig 13), thus offering a promising new approach to treating this debilitating disease (see below).

The partial effects of *P2RX7* ablation observed in G-*mdx*/P2X7^−/−^ mice (which retained one of the P2RX7 isoforms) and in heterozygous *mdx*/P2X7^+/−^ (with intermediate P2RX7 levels) suggest that even incomplete inhibition of this receptor would produce some therapeutic effect. Indeed, treatment with broad purinoceptor antagonists has shown some impact in the *mdx* model of DMD (reviewed in [13]), as did our short-term administration of P2RX7 antagonists (Fig 12). Importantly, the purinoceptor antagonists used here, CBB and ox-ATP, here have slow association rates and do not competitively block agonist activation [50]. We are currently testing new-generation, increased affinity competitive antagonists [51], which are supposed to be significantly more effective, and one such a compound showed some positive effects just after 2 wk of administration (Fig 12).

Regarding the mechanisms by which *P2RX7* ablation produced muscle recovery, these involved both direct effects in dystrophic muscle cells showing over-activation of this receptor [7–11] and reduced inflammation [13,34,52]. Regarding the latter, dystrophin-deficient muscles chronically release high levels of ATP together with other DAMPs, and the resulting immune cell infiltrations contribute to disease progression [34,36]. Targeting innate immunity was shown to slow disease progression [53] and therefore may become a new therapeutic strategy to treat DMD. Deletion of P2RX7, with its unique capability to respond to high eATP levels and to activate inflammation through release of pro-inflammatory cytokines [14], is bound to make a significant impact. Indeed, in *mdx*/P2X7^−/−^ mice, we found an overall decrease in the muscle inflammatory signature and reduced numbers of infiltrating leukocytes. Moreover, we also discovered increased Foxp3 and IL12α mRNA levels in both 4-wk-old and 20-mo-old muscles, indicating a shift in T-cell responses: Foxp3 transcription factor is required for T_reg_ cell development and function, while IL12α is highly expressed by mouse Foxp3^+^ T_reg_ cells but not by effector CD4^+^ T cells [38]. T_reg_ cells modulate the immune response to maintain tolerance to self-antigens, and since peripheral tolerance breakdown has been demonstrated in both DMD and *mdx* [53–56], this result is very encouraging.

In turn, high eATP acting on P2RX7, which is overexpressed in dystrophic muscle cells [7–9], could activate a number of mechanisms, including ERK phosphorylation and ion channel opening, producing activation of signaling cascades. Treatment with apyrase, an ATP-degrading enzyme, reduced intracellular Ca^2+^ levels in *mdx* fibers [57], thus confirming that purinoceptors contribute to the deregulated Ca^2+^ homeostasis in dystrophic muscles. Therefore, *P2RX7* ablation or inhibition would eliminate Ca^2+^ influx occurring via this receptor and also triggering secondary modulation of other Ca^2+^ channels, abnormal functions of which have been described in *mdx* myofibers [58,59].

In addition, we have shown recently that the P2RX7 large pore formation (occurring at high eATP levels) leads to the autophagic death of dystrophic muscle cells [12], which contributes both to fiber loss and to exhaustion of the pool of muscle-resident stem cells required for repair and regeneration [43,60]. Indeed, *P2RX7* ablation reduced autophagy in both 4-wk-old and 20-mo-old *mdx* TA. Further studies are needed to clarify the puzzling shift between positive and negative roles for autophagy described in young and old dystrophic muscles, respectively [61].

The improved heart, leg, and diaphragm phenotype in 20-mo-old *mdx*/P2X7^−/−^ muscles demonstrates that similar mechanisms operate in both skeletal and cardiac muscles and remain active in muscles undergoing continuous, severe degeneration/regeneration akin to human disease. Interestingly, we have confirmed the previous observation [62] that a higher level of fibrosis in diaphragms may coincide with a lower proportion of C/N myofibers (Fig 9). Central nuclei denote muscle regeneration and thus are considered an indicator of muscle pathogenesis. However, as demonstrated in both studies, the proportion of C/N fibers alone is not an accurate marker of muscle degeneration. It has been suggested that such myofibers may be more resistant to mechanical stress [63], which, in turn, could contribute to the differences in the pathology observed in diaphragm compared to limb muscles. The presence of fewer revertant fibers has been used as an indicator that a muscle has undergone fewer degeneration/regeneration cycles [43,44]. Revertant fibers arise due to rare, spontaneous exon skipping events, which take place only in proliferating precursor cells activated by muscle degeneration. Therefore, in experimental paradigms not inducing dystrophin exon skipping, such as described here, revertant fibers are a good indicator of muscles’ degeneration/regeneration history.

The cardiac improvement observed when P2RX7 was blocked is also of clinical importance because, with prolonged patient survival (due to advances in general care), heart failure becomes a more common cause of death in DMD.

Targeting P2RX7 is, to our knowledge, the first clinically relevant treatment for DMD cognitive dysfunction. This is important because severe cognitive impairment occurs in one-third of DMD patients. Absence of dystrophin causes a rearrangement of the precise spatiotemporal pattern of synaptic transmission [64,65]. Re-expression of dystrophin in *mdx* brains results in some improvement [48], indicating that at least partial correction of the cognitive phenotype can be achieved, even in adult brains. However, gene targeting into the CNS is inherently difficult, and therefore currently tested therapies do not address this aspect of the pathology. In contrast, treatments downstream from the primary deficit offer a better chance of success by exploiting more accessible targets. It is currently not known whether dystrophin absence causes P2RX7 abnormalities in brain cells similar to those identified in muscles and DMD lymphoblasts [6]. In fact, it is not unequivocal which brain cells express P2RX7 [66]. Therefore, we are investigating the dystrophic CNS to establish whether it is possible to attribute this improvement directly to P2RX7 ablation in specific cells. An alternative explanation is that the leaky blood–brain barrier found in dystrophic brains [67] causes inflammatory mediators to affect brain functions [68], in which case, the decreased chronic inflammation in *mdx*/P2X7^−/−^ mice would be what results in improved brain performance. Whatever the mechanism, our findings suggest that DMD-associated cognitive impairment could be treated with administration of P2RX7 antagonists. Such drugs, including brain-permeable ones, are undergoing trials for other diseases [69].

P2RX7 plays significantly different roles in bone physiology and in disease states [70]. Importantly, ablation of *P2RX7* in our model did not exacerbate but rather improved the dystrophic bone phenotype. As bone abnormalities in *mdx* mice have been linked to chronic inflammation [71], the reduced inflammatory signature in *mdx*/P2X7^−/−^ muscles may also translate into reduced bone loss in these mice.

A therapeutic strategy that can improve both muscle and non-muscle abnormalities would be a significant development: bone deformities contribute to the physical disability and death of DMD patients. In turn, the brain is the second major site of dystrophin gene expression, and cognitive and behavioral impairments associated with DMD add to the very severe burden of this disease, affecting the quality of life of patients and their families.

The molecular mechanism leading to the P2RX7 abnormality in dystrophinopathy found both in muscle [7–9] and non-muscle cells [6] is unknown. Is this a structural defect related to the scaffolding function of dystrophin or a regulatory defect at the gene or the transcript level? Nonetheless, normal DMD gene expression is essential for normal functioning of various cells, and DMD mutations have detrimental effects evident already at early stages of the disease [72–74]. These findings may lead to a rethink of DMD pathogenesis and treatment.

In summary, loss of dystrophin disrupts many downstream processes. Recent examples have illustrated that such processes may offer good targets for therapeutic interventions that are not constrained by the causative mutation [5,40,52]. Moreover, such approaches may be effective not only in protecting muscle cells but also with respect to inflammation, cognitive impairment, and bone abnormalities, which all make substantial contributions to the DMD pathology.

Our data show that selective ablation or blockade of P2RX7 ameliorates the *mdx* dystrophic process both short and long term and does not cause detectable side effects in this DMD mouse model. Given that specific P2RX7 antagonists have been in human trials for other conditions [75], these could be readily repurposed for treatment of this lethal disease. Furthermore, nucleoside reverse transcriptase inhibitors (e.g., zidovudine) have been shown to block P2RX7 activation in a number of disease models [76]. These drugs, with an extensive pharmacological and safety record for human use, including in children, appear ready for trials in DMD.

## Supporting Information

S1 Checklist The ARRIVE Guidelines checklist. (PDF)

S1 Alternative Language Abstract Arabic translation of the abstract by Rasha Al-Khalidi. (DOCX)

S2 Alternative Language Abstract Chinese translation of the abstract by Taiwen Jiang. (DOCX)

S3 Alternative Language Abstract Italian translation of the abstract by Anna Teti. (DOCX)

S4 Alternative Language Abstract Japanese translation of the abstract by Chikako Yoshida-Noro. (DOCX)

S5 Alternative Language Abstract French translation of the abstract by David Vaudry and Jean-Claude doRego. (DOCX)

S6 Alternative Language Abstract Polish translation of the abstract by D. Gorecki and B. and Z. Zabłocki. (DOCX)

S7 Alternative Language Abstract Russian translation of the abstract by Daria Morgacheva and Mikhail Shugay. (DOCX)

S1 Fig Intermediate levels of muscle P2RX7 correspond with intermediate Feret diameter values in *mdx*/P2X7^+/−^ heterozygous mice. (A) Example Western blots (top) and the average value plot (bottom) illustrating the significant reduction of P2RX7 protein levels in Pf-mdx/P2X7^+/−^ mice compared to *mdx*. Use of separate Western blots is indicated by solid black lines. (B) A frequency histogram of minimum Feret diameter of C/N TA fibers from *mdx*, Pf-*mdx*/P2X7^−/−^, and Pf-*mdx*/P2X7^+/−^ mice showing the intermediate average Feret diameter of TA muscle fibers corresponding with the intermediate level of P2RX7 receptor in these heterozygotes.
(TIF)

S2 Fig Visualization of differences in the expression of genes associated with fibrosis in *mdx* and *mdx*/P2RX7^−/−^ mice. Green—gene down-regulated in Pf-*mdx*/P2X7^−/−^ versus *mdx* mice. Red—gene up-regulated in Pf-*mdx*/P2RX7^−/−^ versus *mdx* mice. Grey—genes present in the dataset but not differentially expressed in *mdx* versus *mdx*/P2RX7^−/−^ mice. Directions of arrows inside the gene symbol indicate up- or down-regulation of this gene in *mdx* versus WT.
(PDF)

S3 Fig Example μCT images and analysis of trabecular morphometry comparing femur bones from 6-mo-old *mdx* with those from WT controls. The proximal femur underwent μCT imaging for the determination of trabecular parameters at 40 kV, 100 μA. With an isotropic voxel size of 5 μm, the image acquisition was performed at a rotational step of 0.19° over 360° for 90 min. The 3-D reconstruction of the samples was obtained using VGStudio Max 2.0 (Volume Graphics). The calculation of the morphometric parameters was carried out by importing the CT images into ImageJ software. A region of interest (ROI) containing trabecular bone only was defined, and for each specimen the following morphometric parameters were determined: BV/TV, trabecular thickness (Tb.Th) and Tb.Sp. Measurements were averaged over ten consecutive slices just below the femoral head.
(TIF)

S1 Table Depiction of the qPCR data comparing relative gene expression levels in gastrocnemius*-*derived mRNAs from WT, *mdx*, Pf-*mdx*/P2X7^−/−^, and G-*mdx*/P2X7^−/−^ mice. Statistically significant differences in ANOVA with Tukey’s post hoc test at *p <* 0.001 are depicted in red and green for up- and down-regulated genes, respectively, and values (2^−ΔΔCT^) shown. Not included were the following genes, where no statistically significant differences in qPCR analyses were found: *Bcl10*, *C1ra*, *C2*, *Casp3*, *Casp9*, *Cd163*, *Cebpa*, *Csf1*, *Cxcr3*, *Cxcr4*, *Fas*, *Fos*, *Foxo3*, *Gpx4*, *Hif1a*, *Hspa1b*, *Hspd1*, *Ifngr1*, *Igf1*, *Il6ra*, *Il10rb*, *Irf1*, *Irf3*, *Irf9*, *Jun*, *Mmp2*, *Nfkb1*, *Nos2*, *P2ry12*, *Prdx5*, *Ptges*, *Rbpj*, *Rplp0*, *Socs1*, *Socs3*, *Sod1*, *Stat1*, *Stat3*, *Tgfbr1*, *Tnfrsf1a*, and *Twist1*. The following genes gave expression values below the detection threshold or ill-reproducible results in repeated experiments: *Adora1*, *Bdnf*, *Ccl5*, *Il1a*, *Il6*, *Il17a*, *Il18*, *Pla2g5*, *Serping1*, and *Tnfrsf1b*.
(PDF)

S2 Table Expression of fibrosis-related and collagen genes estimated using RNA-Seq. Fragments per kilobase per million fragments mapped (FPKM) values as calculated by Cuffdiff and Cuffnorm (Cufflinks suite, http://cole-trapnell-lab.github.io/cufflinks/) are given in the tables cuffdiff_fpkm and cuffnorm_fpkm. The results of the differential expression analysis performed using Cuffdiff are provided in the table cuffdiff_diff_expression.
(XLSX)

## Abbreviations

BV/TV: bone volume/total volume
C/N: centrally nucleated
CBB: Coomassie Brilliant Blue G 250
CK: creatine kinase
CNS: central nervous system
DAMP: danger/damage-associated molecular pattern
DAP: dystrophin-associated protein
DMD: Duchenne muscular dystrophy
eATP: extracellular ATP
ox-ATP: oxidized ATP
TA: tibialis anterior
qPCR: quantitative PCR
TBST: Tris buffered saline with Tween 20
Tb.Sp: trabecular separation
WT: wild-type
μCT: micro-computed tomography
