# Supplementary material for: P2RX7 Purinoceptor: A Therapeutic Target for Ameliorating the Symptoms of Duchenne Muscular Dystrophy
Source: PLoS Med. 2015 Oct 13;12(10):e1001888. doi: 10.1371/journal.pmed.1001888 (PMC4604078; doi:10.1371/journal.pmed.1001888)
Supplement: S1 Alternative Language Abstract — (DOCX) [file pmed.1001888.s002.docx]

**المستقبلات البيورينية: P2rx7 الهدف العلاجي لتخفيف أعراض ضمور العضلات Duchenne muscular dystrophy**

**المقدمة**

مرض Duchenne muscular dystrophy هو احد أكثر الأمراض الوراثية العضلية شيوعا والذي يؤدي إلى العجز الشديد و الموت المبكر للذكور. تعود أسباب الوفاة إلى استمرار عمليات الهدم الحاصلة في العضلات المخططة نتيجة تفاقم الالتهابات. الطفرة الحاصلة بجين الدستروفين تؤثر أيضا على إضعاف القدرات الإدراكية والسلوكية وكثافة العظام.

إلى الوقت الحاضر لا يوجد علاج لهذا المرض. لذلك كل الجهود الممكنة يحب أن تستغل في الكشف عن التغيرات الغير طبيعة المرافقة لغياب جين الدستروفين والذي يعد هدف علاجي مهم. نحن وغيرنا من الباحثين أثبتنا إن الطفرة في جين الدستروفين تغير من مستوى ATP وبالتالي زيادة التعبير الجيني للمستقبلات البيورينية والتي بدورها تعد المسؤولة عن موت العضلات في فثران mdx المستخدمة كنموذج للمرض و في الخلايا المكونة للخلايا اللمفاوية lymphoblast في الإنسان. بالإضافة إلى إن محور ATP-P2rX7 يعد محفز للاستجابة المناعة الطبيعية, فهو يساهم أيضا في استحثاث الالتهابات المزمنة المسؤولة عن أمراضية DMD. لذلك هدفت هذه الدراسة لاختبار مدى تأثير تثبيط P2rX7 في تقليل الصفات المظهرية المرضية في نماذج الفئران لمرض DMD ولمعرفة مدى ملائمتها كهدف علاجي.

**طرق العمل والنتائج**

باستخدام الطرق الجزيئية والهستولوجية والكيموحيوية مع تحليل سلوك حيوانات التجارب, أثبتنا للمرة الأولى إن التعطيل الوراثي للمستقبلات البيورينية P2rx7 في الفئران المستخدمة كنموذج للمرض DMD تنتج عنه تغيرات في الوظائف العضلية وغير العضلية. فقد وجدنا إن العضلات الفاقدة للدستروفين وP2rx7 المأخوذة من الفئران بعمر أربع أسابيع قد تحسنت فيها بعض الوظائف والعوامل الجزيئية مثل هيكل العضلة ( min.Feret diam.P=0.0004 ), زيادة قوة العضلة في الاختبارات in vitro (P=0.0118 ), وأيضا في in vivo (p˂0.0005 ), وانخفاض الالتهابات والجزيئات التي تساهم في التليف. كما تمت ملاحظة انخفاض كل من مستوى أنزيم الكرياتين كاينيز في المصل (P=0.0124 ) والضعف الحاصل بالقدرات العقلية (P=0.0056 ) والتغيرات في تركيب العظم ( P˂0.0005 ). وقد أكدت النتائج تدني في مستوى الالتهابات والتليف عند إجراء الدراسة على الحيوانات بعمر 20 أسبوع أيضا في الساق ( P=0.0382) والحجاب الحاجز (P=0.042) وعضلة القلب (P˂0.0005). تم الحصول على بعض النتائج المشابهة لتعطيل وظيفة P2rx7 بالطرق الوراثية عند استخدام المثبطات الدوائية فمثلا انخفاض مستوى أنزيم الكرياتين كاينيز **( P=0.03 and P=0.0498)** وبدون أثار جانبية. وعلى الرغم من التطبيقات الناجحة للمثبطات الدوائية في حيوانات التجارب إلا إنها مازالت بحاجة إلى المزيد من الاختبارات لإثبات مدى صلاحيتها في العلاج السريري.

الخلاصة

أكدت نتائجنا إن العلاج يمكن أن يحسن من وظائف العضلات على المديين القصير والطويل وكذلك يصلح من ضعف الإدراك وفقدان العظام في الفئران المستخدمة كنموذج لمرضDMD . كما تعكس هذه النتائج الايجابية حقيقة تأثير تثبيط P2rx7 على عدة آليات مرضية كون هذه المستقبلات تتواجد في كل من العضلات الهيكلية والقلبية, والخلايا الالتهابية, العظام والدماغ. وبالتالي تثبيط P2rx7 في فئران المستخدمة كنموذج لمرض DMD يجعل من هذه المستقبلات هدفا للبحوث العلمية: إيجاد دواء أمين لغرض علاج هذا المرض الفتاك.

*Translation: Rasha Al-Khalidi*
