# Supplementary material for: P2RX7 Purinoceptor: A Therapeutic Target for Ameliorating the Symptoms of Duchenne Muscular Dystrophy
Source: PLoS Med. 2015 Oct 13;12(10):e1001888. doi: 10.1371/journal.pmed.1001888 (PMC4604078; doi:10.1371/journal.pmed.1001888)
Supplement: S2 Alternative Language Abstract — (DOCX) [file pmed.1001888.s003.docx]

**P2RX7嘌呤受体：一种可减轻杜氏肌营养不良症状的治疗靶标**

**摘 要**

1、研究背景

杜氏肌营养不良症(DMD)是一种最常见的肌肉遗传病，常导致年轻男性严重残疾和死亡,这种死亡是无菌性炎症引发的严重的横纹肌退行性病变造成的；DMD相关基因突变还可引起认知和行为障碍以及骨密度降低等症状。DMD现有疗法只是姑息治疗，并无长期疗效，因此，有必要寻找更有效的治疗方法。抗肌萎缩蛋白的缺失导致下游关键信号通路异常，而这些异常的信号分子可能是DMD治疗的重要靶标。我们和他人的研究证明DMD相关基因突变能改变ATP信号传导，并且确证P2RX7嘌呤受体上调是DMD的mdx模型小鼠肌肉组织和DMD病人淋巴母细胞死亡的原因。研究还发现，ATP-P2RX7是先天免疫应答激活的关键信号通路，是激发DMD慢性炎症的原因。我们试图通过敲除P2RX7能否缓解DMD小鼠的症状来判断该基因是否为适合的治疗靶标。

2、方法与发现

通过分子生物学、组织学和生物化学方法以及活体行为分析，我们发现在DMD小鼠模型中P2RX7基因的敲除使肌肉和非肌肉症状得到了普遍的缓解，这一发现在国际上尚属首次。在敲除了基因的4周龄DMD小鼠肌肉组织中，一些关键功能和分子参数均有显著改善，如肌肉结构得到改良（min. Feret diam. P=0.0004），体内（P=0.0118）和体外（P<0.0005）试验都表现肌肉强度加大，肌肉炎症降低，促进纤维化分子信号出现。基因敲除后还表现出血清肌酸激酶水平显著降低（P=0.0124），认知损伤得到修复（P=0.0056），骨质结构显著改善（P<0.0005），在腿部肌肉（P=0.0382），膈肌（P=0.042）和心肌（P<0.0005）中，炎症减轻和肌纤维增生可持续到20月龄。我们的研究还表明症状的改善与受体敲除的程度是成正比的，P2RX7拮抗剂的使用能进一步提高疗效（CK，P=0.03和P=0.0498），且未出现不良反应，当然，上述实验结果还需要进一步的临床验证。

3、结论

我们首次发现，在DMD小鼠模型中，用P2RX7基因敲除疗法能短期甚至长期改善肌肉功能，并能使认知损伤和骨质损失得以修复，还对骨骼肌和心肌、炎症细胞、脑和骨骼等多种组织的病理进程有延缓作用。由于在DMD模型中阻断P2RX7能带来上述疗效，因此该受体对于转化医学研究将是一个富有吸引力的治疗靶标，这意味着现阶段一些使用安全的药物，有可能用于治疗DMD这种致命的疾病。

*Translation: Taiwen Jiang*
