# Supplementary material for: P2RX7 Purinoceptor: A Therapeutic Target for Ameliorating the Symptoms of Duchenne Muscular Dystrophy
Source: PLoS Med. 2015 Oct 13;12(10):e1001888. doi: 10.1371/journal.pmed.1001888 (PMC4604078; doi:10.1371/journal.pmed.1001888)
Supplement: S3 Alternative Language Abstract — (DOCX) [file pmed.1001888.s004.docx]

Recettori purinici P2RX7: un bersaglio terapeutico per migliorare i sintomi della distrofia muscolare di Duchenne

Introduzione

La distrofia muscolare di Duchenne (DMD) è la causa più frequente di malattia muscolare ereditaria, la quale provoca disabilità grave e morte in giovani maschi. Il decesso è causato dalla degenerazione progressiva dei muscoli striati aggravata da infiammazione sterile. Gli effetti pleiotropici del gene mutato, distrofina, comprendono anche deficit cognitivi e comportamentali e ridotta densità ossea.

Gli interventi attuali nella DMD sono solo palliativi e nessuno di essi induce miglioramenti persistenti a lungo termine. Pertanto, utilizzando approcci translazionali innovativi si potrebbero identificare anomalie indotte a valle dall’assenza della distrofina, da studiare quali importanti bersagli terapeutici. Alcuni autori, noi compresi, hanno dimostrato che mutazioni nella DMD alterano il segnale dell’ATP ed hanno confermato che l’aumento dell’espressione del recettore purino P2RX7 è responsabile della morte muscolare nel modello murino di DMD, *mdx*, ed in linfoblasti umani DMD. Inoltre, essendo l’asse ATP-P2RX7 un attivatore cruciale della risposta immunitaria innata, esso potrebbe partecipare alla patogenesi della DMD stimolando l’infiammazione cronica. In questo studio abbiamo pertanto verificato se l’ablazione del P2RX7 attenuasse il fenotipo DMD nel modello murino *mdx*, rappresentando così un importante bersaglio farmacologico.

Metodi e risultati

Usando una combinazione di metodi molecolari, istologici e biochimici, unitamente ad analisi comportamentali *in vivo*, abbiamo dimostrato, per la prima volta, che l’ablazione genetica di P2RX7 nel modello murino di DMD produceva una estesa attenuazione funzionale sia dei sintomi muscolari che non-muscolari. Nei muscoli distrofici di topi di 4 settimane si osservava un evidente recupero di parametri funzionali e molecolari chiave, con un miglioramento della struttura del muscolo (min. Feret diam. P=0.0004), un aumento della forza muscolare *in vitro* (P=0.0118) ed *in vivo* (P<0.0005) ed una diminuzione dell’infiammazione e della firma molecolare pro-fibrotica. I livelli di creatin chinasi sierica (CK) erano più bassi (P=0.0124), con un miglioramento del deficit cognitivo (P= 0.0056) e delle alterazioni strutturali delle ossa (P<0.0005). La riduzione dell’infiammazione e della fibrosi era persistente a 20 mesi di età nei muscoli degli arti (P=0.0382), nel diaframma (P=0.042) e nel muscolo cardiaco (P<0.0005). Abbiamo anche osservato che il miglioramento dei sintomi era direttamente proporzionale all’estensione della deplezione del recettore e che esso avveniva anche in seguito a somministrazione di antagonisti di P2RX7 (CK, P=0.03 and P=0.0498,) senza alcun effetto collaterale evidente. Gli approcci efficaci nei modelli animali necessitano però di una prova sperimentale clinica.

Conclusioni

Per quanto a nostra conoscenza, questi risultati sono i primi che stabiliscono che un singolo trattamento possa migliorare la funzione muscolare sia a breve che a lungo termine, e che possa correggere i difetti cognitivi ed ossei nel modello murino di DMD. Il miglioramento generalizzato da noi osservato riflette probabilmente il fatto che l’ablazione di P2RX7 converge su meccanismi patologici multipli che colpiscono i muscoli scheletrici e cardiaci, le cellule infiammatorie, l’encefalo e l’osso. Dato l’impatto positivo del blocco di P2RX7 nel modello DMD, questo recettore può rappresentare un importante bersaglio per la ricerca traslazionale. Farmaci già esistenti e già verificati per la loro sicurezza potrebbero potenzialmente essere riposizionati per il trattamento di questa patologia letale.

*Translation: Anna Teti*
