# Supplementary material for: P2RX7 Purinoceptor: A Therapeutic Target for Ameliorating the Symptoms of Duchenne Muscular Dystrophy
Source: PLoS Med. 2015 Oct 13;12(10):e1001888. doi: 10.1371/journal.pmed.1001888 (PMC4604078; doi:10.1371/journal.pmed.1001888)
Supplement: S4 Alternative Language Abstract — (DOCX) [file pmed.1001888.s005.docx]

**P2RX7プリンノセプター：デュシャンヌ型筋ジストロフィーの症状を改善するための治療標的。**

**要約**

**背景**

**デュシェンヌ型筋ジストロフィー（DMD）は、若い男性に重度の障害と死をもたらす最も一般的な遺伝性筋疾患である。患者は、無菌性炎症により悪化する横紋筋の進行性変性によって死に至る。変異遺伝子の多面的影響により、認知および行動障害、低骨密度も引き起こされる。**

**現状DMDに対する治療は一時しのぎの治療のみで、長期的結果を改善する治療はない。従って、臨床治療への可能性につながるアプローチを検討すべきであり、DMD遺伝子産物であるジストロフィンの欠損からその下流に起こる主要な異常はその強力な治療標的となる。私たちおよび他の研究者らは、DMDの変異がATPシグナリングを変化させることを証明し、P2RX7プリノセプターの発現上昇が、DMDのマウスモデルであるmdxマウスにおける筋肉死およびヒトDMD患者のリンパ芽球死の原因であることを特定した。さらにATP-P2RX7軸は、自然免疫応答の重要な活性化因子であり、慢性炎症を刺激することにより、DMDの病態に寄与している可能性がある。私たちは、レセプターが治療標的として適切かどうかを評価するために、P2RX7の除去によりDMDモデルマウスの表現型が減衰するかどうかについて調べた。**

**方法と結果**

**分子的、組織学的および生化学的方法の組み合わせ、および生体を用いた行動分析を用いて、P2RX7の遺伝子除去によりDMDモデルマウスにおける筋肉および非筋肉症状が広範に機能的に軽減することを、我々は初めて証明した。 4週令のマウスのジストロフィーの筋肉では、筋肉構造の改善(min. Feret diam. P=0.0004)、 *in vitro*（P=0.0118）および*in vivo* (P<0.0005 )における筋肉強度の増強、炎症および前線維性分子兆候の減少など、重要な機能的分子的パラメーターに顕著な回復が見られた。また、血清クレアチンキナーゼ（CK）レベルは低下し（P=0.0124）、認知障害（P=0.0056）および骨構造の変化（P<0.0005）も明らかに減少した。炎症および線維化の減少は、足（P=0.0382）、横隔膜（P=0.042）、および心筋（P<0.0005）において20月令まで継続した。私たちは、症状の改善がレセプター除去の程度に比例し、P2RX7拮抗薬の投与により検出可能な副作用なしに改善が見られること（CK、P=0.03およびP=0.0498）を示した。しかし、動物モデルでは成功したこれらのアプローチが、臨床的実践に効果的かどうかについては、まだ証明する必要がある。**

結論

それにもかかわらず、これらの結果は、単一の処置によって、DMDモデルマウスにおける筋機能が短期的および長期的に改善し、また認知障害や骨損失が矯正されることを、私たちの知る限り、初めて確立したことになる。P2X7除去により幅広い改善が起こることは、骨格筋および心筋、炎症細胞、脳や骨に影響を与える複数の病気のメカニズムがP2RX7に収斂していることを反映している。DMDモデルにおけるP2RX7阻止の影響から、この受容体は、トランスレーショナルリサーチのための魅力的な標的である：確立された安全性記録を持つ既存薬を、この致命的な疾患の治療のために再目的使用できる可能性がある。

*Translation: Chikako Yoshida-Noro*
