# Supplementary material for: P2RX7 Purinoceptor: A Therapeutic Target for Ameliorating the Symptoms of Duchenne Muscular Dystrophy
Source: PLoS Med. 2015 Oct 13;12(10):e1001888. doi: 10.1371/journal.pmed.1001888 (PMC4604078; doi:10.1371/journal.pmed.1001888)
Supplement: S5 Alternative Language Abstract — (DOCX) [file pmed.1001888.s006.docx]

**Le purinorécepteur P2RX7, une cible thérapeutique pour améliorer les symptômes de la dystrophie musculaire de Duchenne.**

Résumé
Contexte
La dystrophie musculaire de Duchenne (DMD) est la maladie musculaire héréditaire la plus fréquente, conduisant à une invalidité sévère et au décès d’hommes jeunes. La mort est causée par la dégénérescence progressive des muscles striés et par des mécanismes inflammatoires. Les effets pléiotropiques du gène muté provoquent également des troubles cognitifs et comportementaux ainsi qu’une faible densité osseuse.

Les traitements actuels de la DMD ne sont que palliatifs puisqu’il n’existe aucun traitement capable d’améliorer la situation à long terme. Par conséquent, de nouvelles approches doivent être étudiées et certaines anomalies observées au niveau moléculaire comme la dystrophine, représentent des cibles thérapeutiques importantes. Nous et d'autres avons démontré que des mutations de la DMD modifient la signalisation de l’ATP et identifié l’activation du purinorécepteur P2RX7 comme étant responsable de la mort de cellules musculaires dans le modèle de souris de la DMD mdx ainsi que dans des lymphoblastes humains de personnes atteintes de DMD. En outre, la voie ATP-P2RX7, étant un activateur clé de la réponse immunitaire innée, elle peut aussi contribuer au développement de la DMD en induisant une réponse inflammatoire chronique. Nous avons donc examiné si l'invalidation du gène de la P2RX7 atténue les effets de la DMD dans un modèle murin de la maladie afin d’évaluer le potentiel de ce récepteur comme cible thérapeutique.

Méthodes et résultats

En utilisant une combinaison d’approches moléculaires, biochimiques et histologiques et des analyses comportementales in vivo, nous avons démontré, pour la première fois, que l'ablation du gène P2RX7 dans un modèle murin de la DMD réduit les symptômes musculaires et non musculaires de la maladie. Dans les muscles dystrophiques chez les animaux âgés de 4 semaines, il y avait une récupération marquée de plusieurs paramètres fonctionnels et moléculaires clés avec par exemple une amélioration de la structure des muscles (P = 0,0004), une augmentation de la force musculaire in vitro (P = 0,0118) et in vivo (P <0,0005 ), et une diminution des marqueurs de l’inflammation et des signatures moléculaires pro-fibrotiques. Les niveaux de kinase créatique (KC ; P = 0,0124), les troubles cognitifs (P = 0,0056) et la structure osseuse (P <0,0005) étaient également améliorés. La réduction de l'inflammation et de la fibrose étaient toujours observées chez les animaux âgés de 20 mois au niveau de la jambe (P = 0,0382), du diaphragme (P = 0,042) et des muscles cardiaques (P <0,0005). Nous avons montré que l'amélioration des symptômes est proportionnelle au niveau de la répression du récepteur et que les améliorations sont observées suite à l'administration d'antagonistes du récepteur P2RX7, sans effets secondaires détectables. Cependant, ces résultats obtenus dans des modèles animaux doivent maintenant prouver leur efficacité en clinique.

Conclusions
Ces résultats sont, à notre connaissance, les premiers à établir qu'un traitement peut améliorer la fonction musculaire à la fois à court et à long terme ainsi que les troubles cognitifs et la perte osseuse dans un modèle de souris DMD. Les améliorations notables reflètent l’importance du récepteur P2RX7 dans les mécanismes affectant les muscles squelettiques et cardiaques, la réponse inflammatoire, le cerveau et les os dans la DMD. Compte tenu des effets bénéfiques du blocage du récepteur P2RX7 dans notre modèle de DMD, ce récepteur apparait comme une cible très intéressante pour le développement de traitements. Enfin, nos travaux montrent que certains médicaments déjà disponibles pourraient être utilisés pour le traitement de cette maladie mortelle.

*Translation: David Vaudry and Jean-Claude doRego*
