# Supplementary material for: P2RX7 Purinoceptor: A Therapeutic Target for Ameliorating the Symptoms of Duchenne Muscular Dystrophy
Source: PLoS Med. 2015 Oct 13;12(10):e1001888. doi: 10.1371/journal.pmed.1001888 (PMC4604078; doi:10.1371/journal.pmed.1001888)
Supplement: S6 Alternative Language Abstract — (DOCX) [file pmed.1001888.s007.docx]

**Receptor purynergiczny P2RX7: cel terapeutyczny łagodzący objawy dystrofii mięśniowej Duchenna**

Tło

Dystrofia mięśniowa Duchenna (DMD) jest najczęściej występującą wrodzoną chorobą mięśni prowadzącą do poważnego inwalidztwa i śmierci młodych mężczyzn. Bezpośrednią przyczyną jest postępujący zanik mięśni prążkowanych w warunkach przewlekłego stanu zapalnego. Ponadto efekty plejotropowe mutacji genu DMD przejawiają się także upośledzeniem poznawczym i obniżoną gęstością mineralną kości.

DMD nie można wyleczyć a obecnie dostępna terapia ma jedynie charakter paliatywny. Dlatego konieczne jest poszukiwanie nowych podejść terapeutycznych o potencjale translacyjnym a zmiany wtórne, nie wynikające bezpośrednio z braku produktu genu DMD (dystrofiny), wydają się być atrakcyjnymi celami terapeutycznymi.

Badania, tak nasze jak i innych wykazały, że mutacjom w genie DMD towarzyszy zwiększenie przekazywania sygnału poprzez receptor purynergiczny P2RX7, co prowadzi do śmierci komórek mięśniowych w mysim modelu choroby (*mdx)* oraz ludzkich dystroficznych limfoblastów. Ponadto oś ATP-P2RX7, będąc kluczowym aktywatorem wrodzonej odpowiedzi odpornościowej, może nasilać patologię DMD pobudzając przewlekły stan zapalny.

W prezentowanej pracy badaliśmy czy wyłączenie P2RX7 zahamuje postępy choroby w mysim modelu DMD by ustalić czy receptor ten jest dobrym celem terapeutycznym.

Metodyka i Wyniki

Stosując kombinację metod molekularnych, histologicznych, biochemicznych oraz badań behavioralnych in vivo wykazaliśmy po raz pierwszy, że genetyczna ablacja receptora P2RX7 w tym modelu DMD powoduje daleko idącą naprawę zmian mięśnowych jak i obserwowanyh w innych tkankach. W mięśniach czterotygodniowych myszy dystroficznych zaobserwowano wyraźną poprawę kluczowych wskaźników funkcjonalnych i molekularnych, w strukturze mięśni (min. średnica Fereta P=0.0004)oraz zwiększoną siłe mięśniową in vitro (P=0.0118) i in vivo (P<0.0005). Wystąpiło także złagodzenie stanu zapalnego i molekularnych wyznaczników zwłóknienia a stężenie kinazy kreatynowej (CK) w surowicy było obniżone (P=0.0124). Ponadto stwierdzono poprawę upośledzenia poznawczego w testach behawioralnych (P= 0.0056) oraz zwiększenie gęstości mineralnej kości (P<0.0005). Redukcja objawów stanu zapalnego i zwłóknienia utrzymywała się w mięśniach kończyn dolnych ((P=0.0382), przepony (P=0.042) i serca (P<0.0005) myszy dwudziestomiesięcznych. Wykazaliśmy również, że złagodzenie tych objawów było proporcjonalne do stopnia obniżenia ekspresji receptora P2RX7. Ponadto, poprawa badanych parametrów obserwowana była również po podaniu antagonistów receptora P2RX7 (CK, P=0.03 i P=0.0498) przy braku zauważalnych objawów ubocznych. Jednakże obiecujące wyniki uzyskane w modelach zwierzęcych wymagają potwierdzenia w praktyce klinicznej.

Wnioski

Według naszej wiedzy, prezentowane wyniki są pierwszym przykładem terapii nie tylko prowadzącej do krótko- jak i długo-terminowej poprawy obserwowanej w mięśniach dystroficznych ale też wpływającej pozytywnie na upośledzenie poznawcze i gęstość mineralną kości. Taka uogólniona poprawa może wynikać z wielofunkcyjności receptora P2RX7, którego wyłączenie bądż zahamowanie wpływają na wiele mechanizmów chorobowych w różnych tkankach: mięśniach szkieletowych i sercu, komórkach układu odpornościowego oraz w mózgu i kościach. Biorąc pod uwagę tak znamienny wpływ zablokowania P2RX7 w mysim modelu DMD, receptor ten jest atrakcyjnym celem terapeutycznym o dużym potencjale klinicznym a istniejące leki o ustalonym poziomie bezpieczeństwa mogłyby być zaadaptowane do terapii tej nieuleczalnej choroby.

*Translation: D Gorecki and B and Z Zabłocki*
