# Supplementary material for: P2RX7 Purinoceptor: A Therapeutic Target for Ameliorating the Symptoms of Duchenne Muscular Dystrophy
Source: PLoS Med. 2015 Oct 13;12(10):e1001888. doi: 10.1371/journal.pmed.1001888 (PMC4604078; doi:10.1371/journal.pmed.1001888)
Supplement: S7 Alternative Language Abstract — (DOCX) [file pmed.1001888.s008.docx]

**P2RX7 пуринорецептор: терапевтическая мишень для улучшения симптомов мышечной дистрофии Дюшенна.**

Введение

Мышечная дистрофия Дюшенна (МДД) — наиболее распространенное наследственное мышечное заболевание, приводящее к тяжелой инвалидности и смерти среди молодых людей. Летальный исход болезни обусловлен прогрессирующей дегенерацией поперечно-полосатых мышц, усугубляемой стерильным воспалением. Плейотропный эффект мутантного гена включает когнитивные и поведенческие нарушения, а также низкую плотность костной массы.

Современные методы лечения МДД являются только паллиативными, так как не способны улучшить состояние в долгосрочной перспективе. Таким образом, существует необходимость в разработке новых подходов с трансляционным потенциалом, и ключевые нарушения вызванные отсутствием продукта гена DMD, дистрофина, выглядят многообещающими терапевтическими целями. Мы и другие исследователи продемонстрировали, что мутации гена DMD могут влиять на сигнальный каскад АТФ, и показали, что повышение экспрессии пуринорецептора P2RX7 ответственно за гибель мышечных волокон в мышиной модели МДД (линия *mdx*) и человеческих лимфобластах при МДД. Более того, взаимодействие АТФ-P2RX7, имеющее решающее значение как активатор врожденного иммунного ответа, может способствовать патологии МДД, стимулируя хроническое воспаление. Для того, чтобы оценить потенциал пуринорецептора P2RX7 в качестве терапевтической мишени, мы проверили, ослабляет ли абляция гена P2RX7 симптомы МДД в мышиной модели.

Результаты и методы

Используя комбинацию молекулярных, гистологических и биохимических методов с анализом поведения мышей *in vivo* мы впервые продемонстрировали, что генетическая абляция P2RX7 в мышиной модели МДД приводит к повсеместному функциональному ослаблению как мышечных, так и не-мышечных симптомо*в.* В пораженных МДД мышцах на 4 неделе наблюдалось заметное восстановление ключевых функциональных и молекулярных параметров. Было показано улучшение структуры мышц (min. Feret diam. P=0.0004), увеличение силы мышц *in vitro* (P=0.0118) и *in vivo* (P<0.0005), уменьшение воспаления и уровня профиброзных молекулярных маркеров МДД. Мы обнаружили снижение уровня сывороточной креатинкиназы (СК) (P=0.0124), уменьшение когнитивных нарушений (P=0.0056) и восстановление структуры костей (P<0.0005). Редукция воспаления и фиброза продолжалась на протяжении 20 месяцев в мышцах ног (P=0.0382), диафрагмы (P=0.042) и сердца (P<0.0005). Мы показали, что улучшение состояния (уменьшение признаков МДД) было пропорционально степени деплеции рецепторов. Положительные изменения также наблюдались при введении антагонистов P2RX7 (CK, P=0.03 and P=0.0498) без каких-либо наблюдаемых побочных эффектов. Однако, методы, успешно опробованные в исследованиях на животных, нуждаются в дальнейшей проверке в клинической практике.

Заключение

Тем не менее, эти результаты, насколько нам известно, впервые устанавливают, что направленное лечение может улучшить мышечную функцию в краткосрочном и долгосрочном периоде, а также скорректировать когнитивные нарушения и разрушения костей в мышиной модели МДД. Широкомасштабные улучшения отражают влияние абляции P2RX7 на множество проявлений заболевания, охватывающих скелетные и сердечные мышцы, воспалительные клетки, мозг и кости. Учитывая сильный эффект блокады P2RX7 в модели МДД, указанный рецептор является привлекательной мишенью для трансляционных исследований: существующие лекарства с установленной безопасностью могут быть перенацелены для лечения этого летального заболевания.

*Translation: Daria Morgacheva and Mikhail Shugay*
