# Supplementary figures and images for: P2RX7 Purinoceptor: A Therapeutic Target for Ameliorating the Symptoms of Duchenne Muscular Dystrophy
Source: PLoS Med. 2015 Oct 13;12(10):e1001888. doi: 10.1371/journal.pmed.1001888 (PMC4604078; doi:10.1371/journal.pmed.1001888)

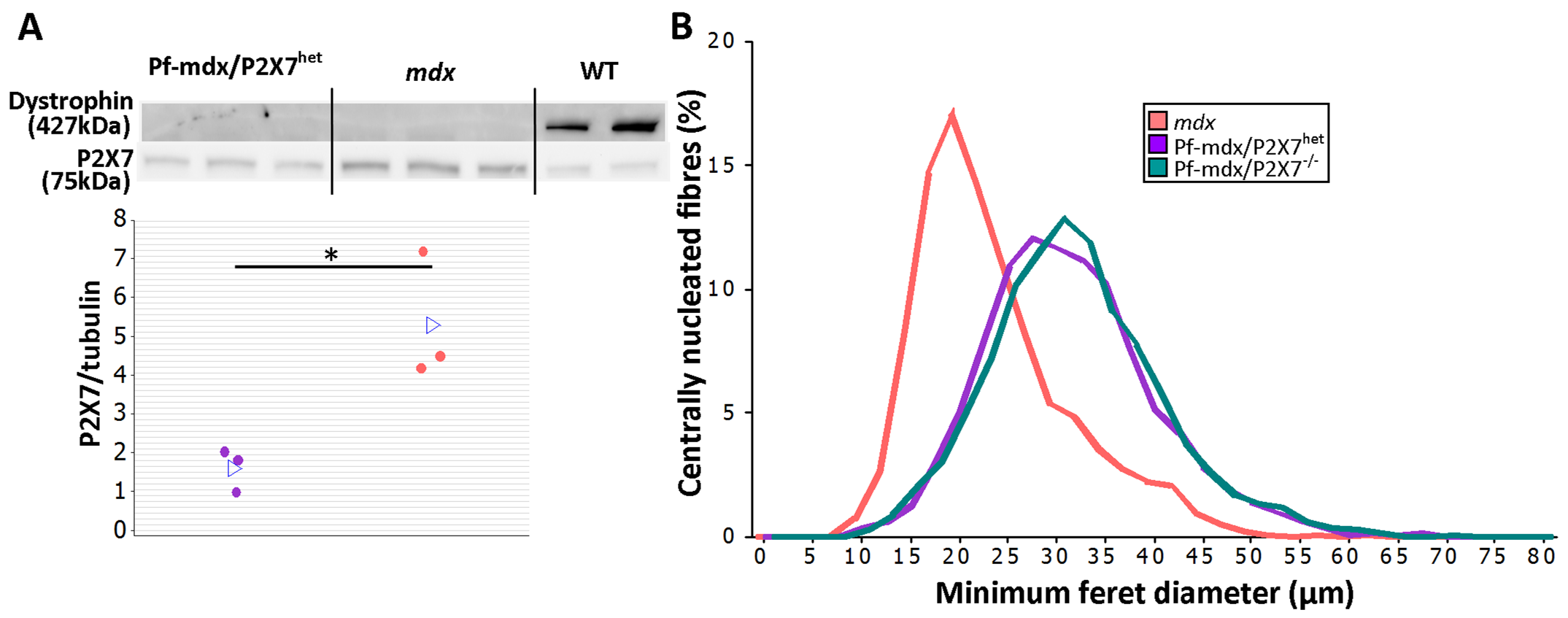

Supplement: S1 Fig — (A) Example Western blots (top) and the average value plot (bottom) illustrating the significant reduction of P2RX7 protein levels in Pf-mdx/P2X7+/− mice compared to mdx. Use of separate Western blots is indicated by solid black lines. (B) A frequency histogram of minimum Feret diameter of C/N TA fibers from mdx, Pf-mdx/P2X7−/−, and Pf-mdx/P2X7+/− mice showing the intermediate average Feret diameter of TA muscle fibers corresponding with the intermediate level of P2RX7 receptor in these heterozygotes. (TIF) [file pmed.1001888.s009.tif]

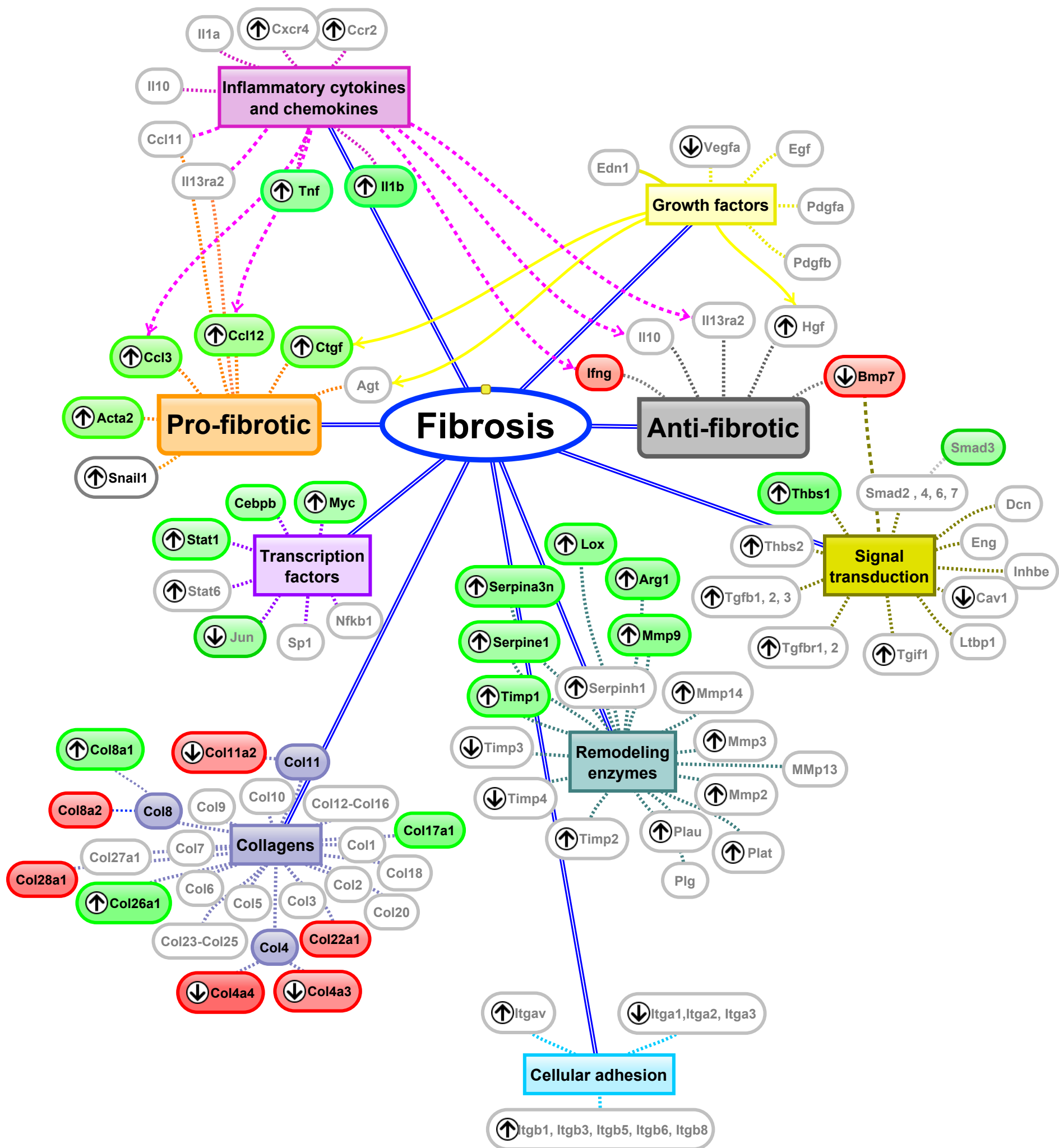

Supplement: S2 Fig — Green—gene down-regulated in Pf-mdx/P2X7−/− versus mdx mice. Red—gene up-regulated in Pf-mdx/P2RX7−/− versus mdx mice. Grey—genes present in the dataset but not differentially expressed in mdx versus mdx/P2RX7−/− mice. Directions of arrows inside the gene symbol indicate up- or down-regulation of this gene in mdx versus WT. (PDF) [file pmed.1001888.s010.pdf]

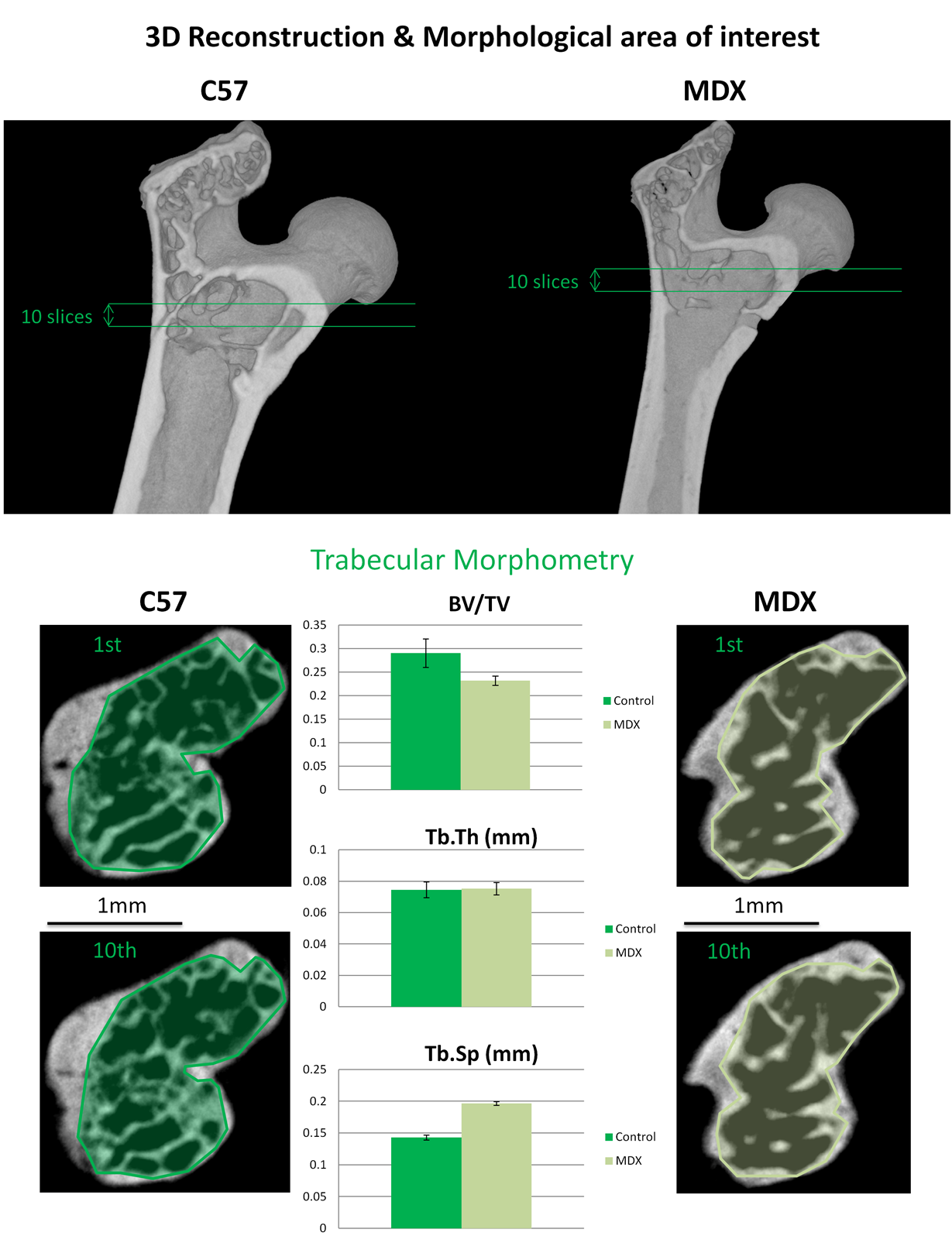

Supplement: S3 Fig — The proximal femur underwent μCT imaging for the determination of trabecular parameters at 40 kV, 100 μA. With an isotropic voxel size of 5 μm, the image acquisition was performed at a rotational step of 0.19° over 360° for 90 min. The 3-D reconstruction of the samples was obtained using VGStudio Max 2.0 (Volume Graphics). The calculation of the morphometric parameters was carried out by importing the CT images into ImageJ software. A region of interest (ROI) containing trabecular bone only was defined, and for each specimen the following morphometric parameters were determined: BV/TV, trabecular thickness (Tb.Th) and Tb.Sp. Measurements were averaged over ten consecutive slices just below the femoral head. (TIF) [file pmed.1001888.s011.tif]
