# Supplementary material for: P2RX7 Purinoceptor: A Therapeutic Target for Ameliorating the Symptoms of Duchenne Muscular Dystrophy
Source: PLoS Med. 2015 Oct 13;12(10):e1001888. doi: 10.1371/journal.pmed.1001888 (PMC4604078; doi:10.1371/journal.pmed.1001888)
Supplement: S1 Table — Statistically significant differences in ANOVA with Tukey’s post hoc test at p < 0.001 are depicted in red and green for up- and down-regulated genes, respectively, and values (2−ΔΔCT) shown. Not included were the following genes, where no statistically significant differences in qPCR analyses were found: Bcl10, C1ra, C2, Casp3, Casp9, Cd163, Cebpa, Csf1, Cxcr3, Cxcr4, Fas, Fos, Foxo3, Gpx4, Hif1a, Hspa1b, Hspd1, Ifngr1, Igf1, Il6ra, Il10rb, Irf1, Irf3, Irf9, Jun, Mmp2, Nfkb1, Nos2, P2ry12, Prdx5, Ptges, Rbpj, Rplp0, Socs1, Socs3, Sod1, Stat1, Stat3, Tgfbr1, Tnfrsf1a, and Twist1. The following genes gave expression values below the detection threshold or ill-reproducible results in repeated experiments: Adora1, Bdnf, Ccl5, Il1a, Il6, Il17a, Il18, Pla2g5, Serping1, and Tnfrsf1b. (PDF) [file pmed.1001888.s012.pdf]

S1 Table; Sinadinos et al.,

Summary of qPCR gene expression analyses.

	Mdx **vs.** WT	Pf-*mdx*/P2X7$^{-/-}$ **vs.** WT	Pf-*mdx*/P2X7$^{-/-}$ **vs.** *mdx*
Apoe	3.298	3.309	1.004
Bax	0.266	0.13	0.491
Bcl2	0.651	0.986	1.515
Bmp7	0.237	0.835	3.526
Ccl2	14.842	7.506	0.425
Ccr5	3.779	3.759	0.995
Cd4	0.26	0.222	1.115
Cx3cr1	9.399	6.064	0.645
Cxcl12	0.627	0.607	0.969
Dmd	0.519	0.520	1.022
Foxp3	4.205	11.153	3.778
Hspb1	0.783	0.888	1.133
Ifng	1.226	2.194	2.01
Il10	3.195	3.516	1.092
IL12a	1.356	1.194	2.041
Il1b	4.205	3.489	0.83
Il6	3.324	3.737	1.335
Mapk14	0.689	0.782	1.135
Mmp9	1.451	3.394	2.339
Myod1	2.024	2.481	1.226
Mrc1	1.585	1.843	1.162
Myd88	1.273	2.603	2.045
Ncf1	2.084	2.991	1.435
P2rx4	4.205	2.825	0.672
P2rx7	6.228	0.066	0.011
P2ry6	2.855	3.37	1.18
Prkaa1	0.626	0.803	1.283
Ptprc	9.377	7.161	0.764
Rela	0.648	0.999	1.52
Rorc	0.577	1.335	2.313
Timp1	2.11	3.557	1.686
Tgfb1	1.934	2.149	1.133
r9	5.415	5.096	0.941
Tollip	0.665	0.636	0.956
Tnf	1.779	0.927	0.512
Trem2	20.07	15.218	0.758
Ucp2	1.94	1.992	1.026
Vegfa	0.437	0.466	1.067
Vegfb	0.577	0.655	1.134
	Pf-*mdx*/P2X7$^{-/-}$ **vs.** G-*mdx*/P2X7$^{-/-}$	G-*mdx*/P2X7$^{-/-}$ **vs.** mdx	Pf-*mdx*/P2X7$^{-/-}$ **vs.** *mdx*
Cx3cl1	1.264	0.493	0.623
Ly6g	0.801	0.597	0.478
Rorc	1.376	0.252	0.347
Tnf	0.603	0.779	0.47
